# Guideline (S2k) on acute therapy and management of anaphylaxis: 2021 update: S2k-Guideline of the German Society for Allergology and Clinical Immunology (DGAKI), the Medical Association of German Allergologists (AeDA), the Society of Pediatric Allergology and Environmental Medicine (GPA), the German Academy of Allergology and Environmental Medicine (DAAU), the German Professional Association of Pediatricians (BVKJ), the Society for Neonatology and Pediatric Intensive Care (GNPI), the German Society of Dermatology (DDG), the Austrian Society for Allergology and Immunology (ÖGAI), the Swiss Society for Allergy and Immunology (SGAI), the German Society of Anaesthesiology and Intensive Care Medicine (DGAI), the German Society of Pharmacology (DGP), the German Respiratory Society (DGP), the patient organization German Allergy and Asthma Association (DAAB), the German Working Group of Anaphylaxis Training and Education (AGATE)

**DOI:** 10.1007/s40629-020-00158-y

**Published:** 2021-01-28

**Authors:** Johannes Ring, Kirsten Beyer, Tilo Biedermann, Andreas Bircher, Matthias Fischer, Thomas Fuchs, Axel Heller, Florian Hoffmann, Isidor Huttegger, Thilo Jakob, Ludger Klimek, Matthias V. Kopp, Claudia Kugler, Lars Lange, Oliver Pfaar, Ernst Rietschel, Franziska Rueff, Sabine Schnadt, Roland Seifert, Britta Stöcker, Regina Treudler, Christian Vogelberg, Thomas Werfel, Margitta Worm, Helmut Sitter, Knut Brockow

**Affiliations:** 1 Department Dermatology and Allergology Biederstein, Technical University Munich, Biedersteiner Straße 29, 80802 Munich, Germany; 2 Department of Pediatrics, Division of Pulmonology, Immunology and Critical Care Medicine, Charité—University Hospital Berlin, Berlin, Germany; 3 Department of Dermatology, University Hospital of Basel, Basel, Switzerland; 4 Clinic for Anaesthesiology, Intensive Care Medicine, Emergency Medicine and Pain Therapy, ALB FILS Hospitals Göppingen, Göppingen, Germany; 5 Department of Dermatology, University Hospital Göttingen, Göttingen, Germany; 6 Department of Anesthesiology and Operative Intensive Care Medicine, Medical Faculty, University of Augsburg, Augsburg, Germany; 7 Dr. von Hauner Children’s Hospital, Ludwig Maximilians University, Munich, Germany; 8 Department of Pediatrics, University Hospital Salzburg, Salzburg, Austria; 9 Department of Dermatology and Allergology, University Medical Center Gießen (UKGM), Justus-Liebig-University Gießen, Gießen, Germany; 10 Center of Rhinology and Allergology, Wiesbaden, Germany; 11 Pediatric Respiratory Medicine, Department of Pediatrics, Inselspital, Bern University Hospital, University of Bern, Bern, Switzerland; 12 St. Marien-Hospital Bonn, Bonn, Germany; 13 Section of Rhinology and Allergy, Department of Otorhinolaryngology, Head and Neck Surgery, University Hospital Marburg, Philipps-University Marburg, Marburg, Germany; 14 Department of Pediatrics, University Hospital Cologne, Cologne, Germany; 15 Department of Dermatology and Allergology, Hospital of the Ludwig Maximilians University, Munich, Germany; 16 German Allergy and Asthma Association, Mönchengladbach, Germany; 17 Institute of Pharmacology, Hannover Medical School, Hannover, Germany; 18 Medical practice for pediatrics and youth medicine, Poppelsdorfer Allee, Bonn, Germany; 19 Department of Dermatology, Venereology, and Allergology, Leipzig Interdisciplinary Allergy Center, University Hospital Leipzig, Leipzig, Germany; 20 Department of Pediatric Pneumology and Allergology, University Hospital Carl Gustav Carus, Technical University of Dresden, Dresden, Germany; 21 Immunodermatology and Experimental Allergology Unit, Department of Dermatology, Allergology, and Venereology, Medical University Hannover, Hannover, Germany; 22 Department of Dermatology, Venereology, and Allergology, Charité—University Hospital Berlin, Berlin, Germany; 23 Institute for Surgical Research, Philipps-University Marburg, Marburg, Germany

**Keywords:** Anaphylaxis, Food allergy, Drug allergy, Adrenalin, Emergency management, Pharmacotherapy, Group education, Auto-injector, COVID-19, Vaccination

## Background

Anaphylaxis is an acute systemic reaction involving symptoms of an immediate-type allergic reaction that can comprise the whole organism and potentially be fatal [1–3].

Although anaphylaxis is a highly acute process in terms of its symptoms, there is a chronic immunological imbalance underlying this condition that leads to immediate reactions as soon as contact with the elicitor occurs. This chronic condition may have severe effects of both a psychological and an organizational nature on the everyday life of affected individuals.

The definition of anaphylaxis is not internationally standardized. Also, a number of different classification systems are in use. The most commonly used classification in German-speaking countries is used in this guideline.

Anaphylactic reactions are among the most severe, potentially life-threatening, and dramatic events in allergology. Acute treatment is carried out according to international guidelines and recommendations in textbooks. This guideline is an update of earlier versions from 1994, 2007, and 2014 [4–8] and takes international guidelines [3, 7] into consideration (see Addendum).

Anaphylactic reactions may spontaneously cease at any stage of symptoms, but may also progress in severity despite adequate treatment. This unpredictability makes it difficult to evaluate the efficacy of therapeutic procedures. Single-case observations do not provide evidence-based information on whether specific treatments have been effective.

It is well known that patients, e.g., after successfully treated anaphylaxis due to an insect sting, are not optimally followed-up [9–11]. These problems in basic management underline the need for further research, as well as the importance of the guideline presented here.

This guideline is intended for all physicians, as well as other individuals active in health care, involved in the acute treatment, diagnostics, and management of patients with anaphylaxis.

## Epidemiology of anaphylaxis

In recent years, a number of studies on the worldwide prevalence of anaphylactic reactions have been published [12–21]. Due to the varying definitions, as well as the fact that anaphylaxis with fatal outcome is not always diagnosed, one needs to assume a certain number of unreported cases.

A limitation with regard to data on the epidemiology of anaphylaxis arises due to the variable coding of anaphylaxis according to ICD-10. There are several ICD-10 codes that can include anaphylaxis. The new ICD-11 will be introduced in 2022, possibly with a new classification of anaphylaxis [22–24]. There is a particular need for classification regarding whether repeated cutaneous reactions in manifest type I allergy can already be regarded as anaphylaxis, whether, by definition, involvement of at least two organ systems is required, or whether involvement of only the respiratory and/or cardiovascular system can represent a severe reaction and thus be classified as anaphylaxis. There is currently no national or international consensus on this question. Published data regarding epidemiology need to be evaluated taking these aspects into consideration [25, 26].

Retrospective studies show that up to 1–2% of patients in an emergency unit at a primary care hospital (maximal care) present due to anaphylactic reactions [18]. The number of anaphylaxis-related fatalities is estimated to be between one and three cases per year per million population [19]. Recent studies from the US, UK, and Australia show incidence rates of anaphylaxis of between 7–50/100,000 per year and show an increased incidence of anaphylaxis in recent decades. In particular food-induced anaphylaxis in children and drug-induced anaphylaxis in adults have increased, although the mortality rate has remained unchanged [19–21].

Data from the anaphylaxis registry of German-speaking countries, as well as data from other countries around the world, show that foods are the most common elicitors of anaphylaxis in childhood [26]. Insect venoms and drugs are the most frequent elicitors in adults (Table 1); however, there are international differences with regard to this ranking. In childhood, boys are more often affected by anaphylaxis than are girls, possibly due to the more frequent occurrence of food allergies in boys; these differences between sexes disappear after puberty [27].

**Table 1:** Common elicitors of severe anaphylactic reactions in children and adults (data from the anaphylaxis registry March 2017, *n* = 8046 [28, 29])

Elicitors	Children (in %)	Adults (in %)
Foods	60	16
Insect venoms	22	52
Drugs	7	22
Others	5	3
Unknown	7	6

## Pathophysiology

Anaphylaxis is usually caused by an immunological reaction—most frequently an immunoglobulin E (IgE)-mediated allergy. IgE activates mast cells and basophils via cross-linking of high-affinity IgE receptors, leading to increased expression of surface markers (CD63, CD203c), as indirectly measurable on basophils. The symptoms of anaphylactic reactions are mediated by a variety of substances released from mast cells and basophil granulocytes such as histamine, prostaglandins, leukotrienes (LTB 4, LTC 4, and LTD 4), tryptase, platelet-activating factor (PAF), heparin, proteases, serotonin, and cytokines [30–34]. The relative importance of these mediators in humans cannot easily be estimated for methodological reasons and is a matter of discussion. There is consensus that histamine is involved in anaphylactic reactions [30]. Thus, the intravenous application of histamine can elicit anaphylactic symptoms in healthy individuals [35, 36]. Furthermore, there is discussion as to whether, in addition to IgE in rare cases (e.g., dextran 4,5), other antibody classes can also elicit similar symptoms to, or aggravate, an IgE-mediated reaction; the complement split products, C3a, C4a, and C5a (anaphylatoxins), are the most important mediators in this context and, in addition to basophils, also neutrophils and macrophages play a role as relevant effector cells that can be activated via immune complex receptors (CD16, CD32, or CD64) [37, 38].

Furthermore, there are anaphylactic reactions in which no immunological sensitization can be detected. These reactions are referred to as “pseudo-allergic reactions” [37] or “non-immunological anaphylaxis” [1]. The mechanisms of this non-allergic anaphylaxis include: IgE-independent release of vasoactive mediators, possibly via MAS-related G protein-coupled receptor [39]; direct activation of the complement system; interactions with the kallikrein–kinin system; interactions with arachidonic acid metabolism; as well as psychoneurogenic reflex mechanisms. The state of knowledge of the pathophysiology of these reactions is undoubtedly less well established than for allergic anaphylaxis.

Anaphylaxis can be particularly severe in patients with increased basal serum tryptase levels and/or mastocytosis [40–44]; however, normal tryptase levels have often been measured, especially in children with food-induced anaphylaxis [45]. Previous use of beta-adrenoceptor antagonists and angiotensin converting enzyme (ACE) inhibitors can intensify the severity of anaphylactic symptoms [27, 28, 44, 46].

In the case of beta-adrenoceptor antagonists, a blockade of the cardiostimulatory and mast cell-stabilizing effects of adrenalin play a role, while ACE inhibitors reduce bradykinin metabolism, resulting in increased vasodilatation. The use of cyclooxygenase inhibitors (non-steroidal anti-inflammatory drugs, NSAID) can also cause increased production of leukotrienes, as well as facilitated absorption of orally ingested allergens, thereby leading to increased anaphylactic symptoms.

## Clinical symptoms

Anaphylactic reactions manifest mainly on the skin, as well as in the respiratory tract, gastrointestinal tract, and cardiovascular system. The working group discussed whether the guideline should use a classification of severity grades, since current treatment is performed according to the symptoms of anaphylaxis. The majority favored a severity classification. There are various classifications of anaphylaxis severity in the literature [7, 8, 10, 29], each classification having advantages and disadvantages. The group decided on a modification of the currently most frequently used classification in Germany, which was also used in the previous guideline [5, 6]. According to the intensity of clinical symptoms, anaphylaxis can be classified into severity grades I–IV (Table 2).

**Table 2:** Severity scale for the classification of anaphylactic reactions (modified from [6, 47]). The classification is made according to the most severe symptoms observed (no symptom is mandatory)

Grade	Skin and general subjective symptoms	Abdomen	Respiratory tract	Cardiovascular
*I*	Itch	–	–	–
	Flush			
	Urticaria			
	Angioedema			
*II*	Itch	Nausea	Rhinorrhea	Tachycardia (increase by ≥20/min)
	Flush	Cramps	Hoarseness	
	Urticaria	Vomitus	Dyspnea	Hypotension (decrease by 20 mm Hg systolic pressure)
	Angioedema			
				Arrhythmia
*III*	Itch	Vomiting	Laryngeal edema	Shock
	Flush	Defecation	Bronchospasm	
	Urticaria		Cyanosis	
	Angioedema			
*IV*	Itch	Vomiting	Respiratory arrest	Cardiac arrest
	Flush	Defecation		
	Urticaria			
	Angioedema			

The symptoms of anaphylactic reactions are mostly of acute onset and can progress rapidly. Within minutes, symptoms can intensify and lead to shock and death. However, the reaction can also cease spontaneously at any stage and resolve. With a reaction of grade I severity, the further development and dynamics of the reaction cannot be predicted. The symptoms may vary and occur simultaneously or sequentially. There may be primary cardiovascular reactions without preceding cutaneous or pulmonary symptoms. In 5–20% of affected individuals, a protracted or biphasic clinical course may develop following successful treatment, with renewed symptoms after 6–24 h [48–50]. In addition to acute symptoms immediately after allergen contact and a biphasic course, there are also initially delayed anaphylactic reactions, whereby the symptoms only occur some hours after exposure. These particular kinetics have been well documented, for example, for the allergen galactose-alpha‑1,3‑galactose in mammal meat allergy (“red meat”) and are most likely due to a delayed release or systemic availability of the allergens or their binding sites [51–53]. However, also in peanut allergy, the median time from consumption to symptom onset is 55 min [54].

At the start of an anaphylactic reaction, symptoms may be observed as “prodromal symptoms” with mild itch or a burning sensation on the palms and soles or in the anogenital area, metallic taste, anxiety, headache, and disorientation. Young children are unable to adequately report these symptoms; they often exhibit agitation and withdrawal behavior as initial symptoms prior to objective signs.

Itch, erythema (flush), urticaria, and angioedema (Quincke’s edema) develop on the skin and mucous membranes, also on skin areas that have not come into direct contact with the elicitor (systemic reaction). The skin is the organ most often affected in anaphylaxis.

In the upper airways, patients often report a burning, prickling, or itching sensation on the tongue or palate as initial signs. Swelling of the uvula and tongue can be observed in the oropharynx. Clinical signs include hoarseness or muffled speech, difficulty in swallowing, with salivation or inspiratory stridor. Laryngeal edema can potentially rapidly lead to obstruction of the upper airways and life-threatening hypoxia.

In the lungs, bronchoconstriction and dyspnea may develop, especially in patients with bronchial asthma. Clinical signs include wheezing, prolonged expiration, and tachypnea. Bronchial obstruction is the main symptom in life-threatening reactions, especially in children and adolescents. Here, the severity of asthma correlates directly with the severity of anaphylaxis. Varying degrees of vasoconstriction may develop in some cases, with an extreme increase in pulmonary vascular resistance occurring, sometimes leading to respiratory arrest and need for resuscitation. Pulmonary edema may occur as a result of the impaired permeability [55–57].

Gastrointestinal symptoms include partly cramp-like abdominal pain, nausea, vomiting, and diarrhea. Furthermore, one may see increased intestinal motor activity involving meteorism, urge to defecate, and even involuntary defecation. Other abdominal symptoms can include urge to urinate or micturition, as well uterine cramps. In children, mild oral symptoms or perioral redness with vomiting may be the only symptoms of food-induced anaphylaxis.

As a result of vasodilatation and impaired permeability, there is a loss of fluid into the tissues, leading to hemoconcentration and intravascular hypovolemia, followed by arterial hypotension and tachycardia. Direct cardiac symptoms such as arrhythmia or bradycardia may occur.

Symptoms of the central nervous system include restlessness, withdrawal behavior, headache, cerebral spasms, impaired consciousness, or loss of consciousness. A change in behavior is often observed in children, manifesting as anxiety or sometimes also aggressiveness. Older children, adolescents, and adults may experience a “sense of impending doom.”

If an anaphylactic reaction occurs during general anesthesia, the patient is unable to report early symptoms such as itch or nausea. If observed, erythema, urticaria, or cardiovascular reactions (tachycardia or hypotension), as well as changes in bronchoconstriction affecting ventilation (increased airway resistance, decreased expiratory flow), are particularly significant [58].

Causal factors of lethal anaphylaxis include airway obstruction and/or cardiovascular failure, either as a direct effect on the heart or as a sequela of impaired microcirculation with shock; disseminated intravascular coagulation or adrenaline overdose have been observed in rare cases [59, 60].

## Allergens and elicitors

The most common elicitors of severe anaphylactic reactions include drugs, insect venoms, and foods. The ranking of these elicitors is determined by various factors, such as mode of detection, age group, and geographic region. In German-speaking countries, elicitors of anaphylactic reactions have been registered since 2006 in an “Anaphylaxis Registry,” where allergy centers in Germany, Austria, and Switzerland, as well as other European countries, report cases of severe allergic reactions. In children, foods are the most common elicitors of severe anaphylactic reactions, whereas insect venoms and drugs are common elicitors in adults [25]. The anaphylaxis registry also makes it possible to promptly identify very rare elicitors of anaphylaxis—most recently in foods, e.g., spices or new exotic fruits [28].

Contact with the elicitor of anaphylaxis classically occurs via oral or parenteral (hematogenous) exposure. In rare cases, anaphylaxis can also be elicited via airborne contact or, even more rarely, via skin contact (contact anaphylaxis) in highly sensitized individuals [61–63]. Anaphylactic symptoms may also occur depending on a combination of various factors, e.g., allergen exposure together with physical exercise, known as exercise-induced anaphylaxis (EIA) [64, 65], alcohol, mental or emotional stress, infection, or simultaneous exposure to other allergens, or use of anaphylaxis-inducing drugs. This phenomenon is referred to as augmentation or summation anaphylaxis. A more common form is food-dependent exercise-induced anaphylaxis (FDEIA), which is most frequently elicited by wheat or subspecies such as spelt, green spelt, or emmer [65, 66].

## Risk factors of severe anaphylaxis

**Fig. 1:**
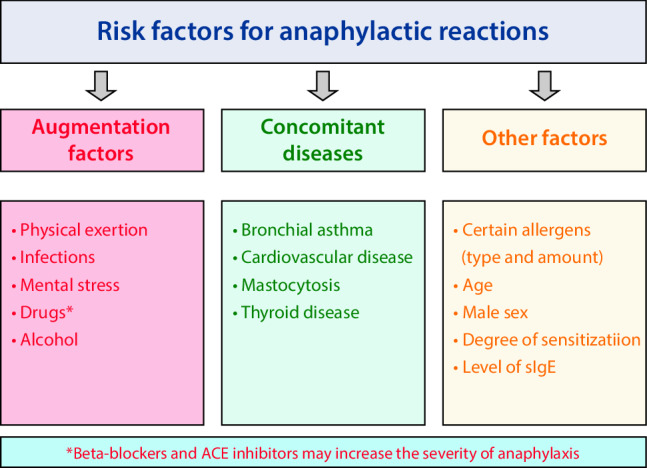
Risk factors for anaphylactic reactions

Certain endogenous or exogenous factors can increase the risk of severe anaphylaxis. Risk factors of this kind (Fig. 1), which are independent of the elicitor, include advanced age, severe cardiovascular disease, (inadequately treated) bronchial asthma, use of certain drugs that promote mast cell activation or leukotriene secretion (such as NSAID), and mastocytosis [28, 41, 67].

Evidence for an increased risk of severe anaphylaxis under medication with beta-adrenoceptor antagonists (beta-blockers) is based on a number of case reports and case series [68–70], as well as two case control studies on anaphylaxis frequency and severity after administration of radiographic contrast media [71, 72]. Recent data from the European anaphylaxis registry confirm that the use of beta-adrenoceptor antagonists is associated with an increased risk for severe anaphylaxis (odds ratio [OR] 1.86) [28].

Considering elicitor-dependent subgroups of anaphylaxis, there are reports of food-induced anaphylaxis showing that allergic bronchial asthma is a major risk factor [73]. Finally, the elicitor itself may be a risk factor—it is known that primary sensitization to peanut or fish, both highly potent allergens, represents a risk factor per se for severe reactions [74].

## Diagnosis and important differential diagnoses

Since the clinical symptoms of anaphylaxis are not always characteristic, diagnosis may be challenging. Therefore, it is important to differentiate other acute reactions from symptoms of anaphylaxis, such as other manifestations of isolated urticaria, bronchial obstruction, vomiting, nausea, diarrhea, agitation, loss of consciousness, cardiac arrhythmia, and/or cardiac arrest. Relevant differential diagnoses are listed in Table 3. After appropriate acute treatment, it is helpful to determine mediators in blood, in particular serum tryptase—ideally 1–3 h after the onset of anaphylaxis and, if possible, as compared to basal serum tryptase. Tryptase can also be determined retrospectively—even post mortem—but is not necessarily elevated [41, 45, 67].

**Table 3:** Important differential diagnoses of anaphylaxis

*Cardiovascular diseases*	Vasovagal syncope
	Cardiogenic shock
	Cardiac arrhythmia
	Hypertensive crisis
	Pulmonary embolism
	Myocardial infarction
	Hemorrhagic shock
	Aortic dissection
	Tension pneumothorax
*Endocrinological diseases*	Carcinoid syndrome
	Pheochromocytoma
	Thyreotoxic crisis
	Hypoglycemia
*Neuropsychiatric diseases*	Hyperventilation syndrome
	Anxiety/panic attacks
	Dissociative disorders and conversion (e.g., globus hystericus)
	Psychosis
	Artefacts (Muenchhausen syndrome)
	Somatoform disorders (e.g., psychogenic dyspnea, vocal cord dysfunction)
	Epilepsy
	Coma, e.g., metabolic, traumatic
*Airway diseases*	Acute severe asthma (without involvement of other organs)
	Acute stenosing laryngotracheitis (croup episode)
	Tracheal/bronchial obstruction (e.g., foreign body)
*Skin diseases*	Urticarial diseases and hereditary/acquired angioneurotic angioedema
Note: In physical urticaria, intensive contact with the elicitor may also give rise to anaphylaxis	
*Pharmacological/toxic reactions*	Ethanol
	Histamine intoxication, e.g., in fish poisoning (scombroid)
	Opiates (morphine)
	Hoigné’s syndrome

The following symptoms are considered to be characteristic criteria for anaphylaxis [8]:

1. Sudden onset of skin symptoms (e.g., acute urticaria, angioedema, flush, mucosal edema) together with sudden respiratory symptoms (e.g., dyspnea, wheezing, cough, stridor) or a sudden decrease in blood pressure (manifesting as, e.g., collapse, tachycardia, incontinence)
2. Sudden onset of symptoms in two or more organ systems: skin (e.g., acute urticaria, angioedema, flush, mucosal edema), gastrointestinal tract (e.g., abdominal cramps, vomiting), respiratory tract (e.g., dyspnea, wheezing, cough, stridor), or cardiovascular system (e.g., decreased blood pressure, collapse, incontinence) after contact with a likely allergen or anaphylactic trigger
3. Drop in blood pressure after contact with an allergen known to the patient or another anaphylaxis trigger

## Pharmacology of the most important drugs in the treatment of anaphylaxis

The following substances have proven to be effective in the pharmacological treatment of anaphylaxis.

### Vasoactive substances

#### Adrenaline (epinephrine)

The most important drug in the acute treatment of anaphylaxis is adrenaline (epinephrine) [75, 76]. By activating alpha- and beta-receptors, adrenaline functionally antagonizes all relevant pathomechanisms of anaphylaxis via vasoconstriction, reduction of vascular permeability, bronchodilatation, reduction of edema, and positive inotropy of the heart. When administered intravenously or intramuscularly, it has the fastest onset of action of all anaphylaxis drugs.

In patients not requiring resuscitation, immediate intramuscular administration of adrenaline at a dose of 0.15–0.6 mg to the outside of the upper thigh is the pharmacological treatment of first choice. The risk of severe cardiac side effects is considerably lower compared to intravenous administration. In the absence of an effect, and depending on adverse events, the injection can be repeated every 5–10 min subject to clinical symptoms.

The subcutaneous injection of adrenaline is no longer recommended due to its insufficient absorption and resulting delayed action.

If symptoms fail to stabilize and circulatory or respiratory decompensation is imminent, adrenaline should be given intravenously [77]. To this end, a dilution of 1 mg adrenaline in 100 ml NaCl 0.9%, i.e., a solution of 10 µg/ml titrated with single boluses of 1 µg/kg body weight (BW), is used under continuous monitoring of circulatory parameters depending on effects and side effects.

Electrocardiogram (ECG), pulse, and blood pressure monitoring is required (see below for adrenaline dosing in cardiac and circulatory arrest). In patients receiving beta-adrenoceptor antagonist therapy and failing to respond to several doses of adrenaline or other vasoactive substances (see below), administration of glucagon is recommended since this has a positive inotropic effect and leads to the up-regulation of beta-adrenoreceptors on the cell surface [78]. However, glucagon only has an effect on cardiac symptoms.

In addition to its intramuscular administration, adrenaline can also be given by inhalation in the case of laryngeal edema and is also effective in bronchospasm. Here, the administration of adrenaline, undiluted (e.g., 3–5 ml at a concentration of 1 mg/ml) via a nebulizer using a breathing mask/mouthpiece together with oxygen is recommended. The inhaled administration of adrenaline does not replace parenteral administration and should only be used in an additive capacity [58].

If bronchial obstruction is the major symptom, the additional administration of inhaled beta-adrenoceptor agonists is effective, e.g., salbutamol, at an initial dose of two puffs—if ineffective, between four and eight puffs, and/or subcutaneous terbutaline. In the case of young children, the efficacy of inhaling a dosed aerosol can be increased by using a “spacer,” together with a mask if required.

In the past, ephedrine was recommended instead of adrenaline for hypotension during pregnancy. However, the evidence for ephedrine is even more scant than for adrenaline; therefore, in line with the recommendations of other authors, the authors recommend the administration of adrenaline also in anaphylaxis during pregnancy [79].

Even when administered appropriately, adrenaline is not always effective and therapeutic failure or side effects may be observed. The increase in cardiac output results in increased oxygen consumption and cardiac muscle necrosis. Adrenaline can also have arrhythmogenic effects; therefore, in patients with pre-existing coronary disease, intravenous adrenaline may cause angina pectoris or myocardial infarction. Although there is no absolute contraindication for adrenaline in severe life-threatening anaphylaxis, the indication should be carefully considered in patients with cardiovascular disease. In the case of asystolic cardiac arrest or pulseless electrical activity on ECG, 1 mg i.v. adrenaline is given every 3–5 min in adults or 0.01 mg/kg in children [80, 81].

#### Other vasoactive substances

Dopamine, noradrenaline, and vasopressin are used in the emergency setting by emergency physicians, as well as under intensive care conditions using cardiopulmonary motoring.

##### Dopamine

Dopamine, which acts on alpha- and beta-adrenoceptors and has a short half-life [82, 83], is no longer used in German emergency and intensive care medicine since it can elicit undesired tachycardia and is markedly less effective in stabilizing blood pressure than adrenaline or noradrenaline, which can be well titrated with syringe drivers.

##### Noradrenaline

Since noradrenaline is a highly potent alpha- and somewhat less potent beta‑1 adrenoceptor agonist and has a lower stimulatory potency at the beta2-adrenoceptor compared to adrenaline, its bronchodilatory effect is lower at therapeutic doses. Therefore, its principal effect is an increase in peripheral resistance and systolic blood pressure. Its effect on the lungs is comparatively small. Noradrenaline is used particularly when volume replacement and adrenaline have an insufficient effect [76, 84]. Due to its marked vasoconstrictive effects, it should be used only as a continuous intravenous infusion under strict blood pressure and pulse monitoring. Dosage is 0.02–0.15 µg/kg per minute.

##### Vasopressin

The use of vasopressin in the treatment of severe hypotension has been described by anesthetists [85].

There are individual reports on the successful use of vasopressin in volume- and catecholamine-refractory shock. This is not an evidence-based treatment; it is optional in extremely severe situations of persistent shock when treatment with volume and other catecholamines has failed. An effect on mortality or duration of intensive care hospitalization could not be shown for children. Dosage is 0.01–0.03 international units (IU)/min.

### Oxygen

**Fig. 2:**
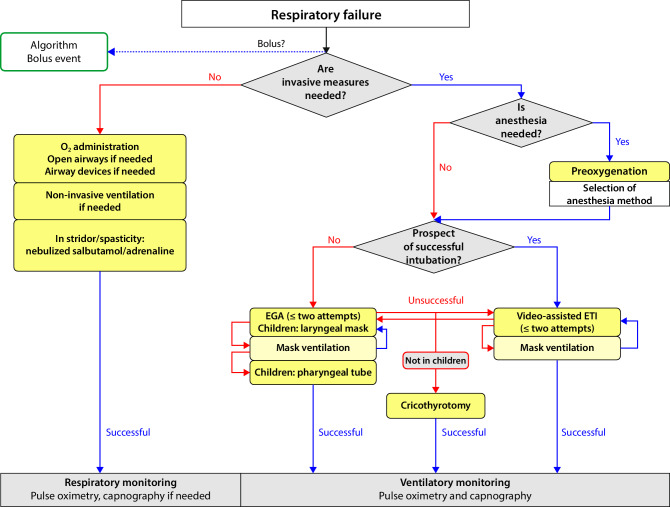
Algorithm for prehospital airway management (from [58]). *EGA* epiglottic airway, *ETI* endotracheal intubation

In manifest cardiovascular or pulmonary reactions, the administration of oxygen via a breathing mask is recommended, in particular a non-rebreather mask. The administration of high-flow oxygen (100%) is recommended. A laryngeal mask or tube may be helpful. Only on rare occasions is tracheal intubation by an experienced physician (usually an emergency physician or anesthetist) necessary. The reader is referred here to the S1 guidelines for prehospital airway management, which provide an algorithm explaining both the indication for and the performance of invasive prehospital airway management (Fig. 2; [58]).

### Volume replacement

A major pathophysiologic aspect of anaphylaxis is the resulting relative hypovolemia induced by vasodilatation and capillary leakage [86]. As such, it is clear that volume therapy can only be used in addition to the crucial mast cell-stabilizing and vasoconstrictor effect of adrenaline therapy [87–89]. This can only be achieved with a large-lumen intravenous catheter. If it is not possible to perform an intravenous injection, intraosseous access needs to be obtained. Anaphylactic shock in adults requires rapid administration of a high amount of volume: 1–3 l of balanced electrolyte solution depending on response. In children, 20 ml/kg BW are initially administered by hand as rapidly as possible. After re-evaluation, repeated boluses of 20 ml/kg are administered until hemodynamic stabilization is achieved.

Gelatin and dextran solutions—despite their positive hemodynamic effects—should not be used in anaphylaxis due to their histamine-releasing potency and their own risk of inducing anaphylaxis (e.g., in the case of dextran without pretreatment with low molecular hapten-dextran) [7].

According to the most recent evaluation by the European Medicines Agency (EMA), hydroxyethyl starch (HES) preparations are contraindicated in the critically ill [90–92]. Due to the lack of relevant literature, the guideline group is somewhat reluctant to make recommendations.

### Antihistamines (histamine H1-receptor antagonists)

The central role of histamine as a mediator of allergic reactions and the effect of histamine H1-receptor antagonists in acute urticaria or rhinoconjunctivitis are undisputed; however, their effects on circulation and bronchoconstriction have not been demonstrated [93]. Antihistamines have a slower onset of action compared to adrenaline, but have a good benefit–risk profile and a wide therapeutic window. One can assume an effect on allergic reactions. Therefore, antihistamines should be given in all anaphylactic reactions in order to antagonize the effect of histamine as early on as at the initial stage, once vital functions have been stabilized. Under no circumstances should immediate life-saving measures such as intramuscular administration of adrenaline, volume replacement, or oxygen administration be delayed by the use of antihistamines!

In terms of intravenous administration in the acute treatment of anaphylaxis, only the first-generation histamine H1-receptor antagonists dimetindene (0.1 mg/kg BW) and clemastine (0.05 mg/kg BW), with their well-known sedative side effects, are available. At higher doses, antihistamines may show antimuscarinic effects ranging from tachycardia, mouth dryness, intestinal atony, urinary retention, increased intraocular pressure to glaucoma attack and paradoxical states of arousal [94]. Therefore, these symptoms need to be borne in mind.

Second-generation histamine H1-antagonists are not approved as yet for the treatment of anaphylaxis and are not available for intravenous injection; nevertheless, the newer, more selective histamine H1-antagonists are often recommended as an oral treatment, having shown rapid onset of action in placebo-controlled skin test studies [93]. In the case of oral antihistamine administration, the maximum approved dose is primarily recommended. However, the expert group agrees that higher doses (up to a maximum of four times the approved single dose) can be given in individual cases, as recommended in the treatment of chronic urticaria [95]. Further studies with newer H1-receptor antagonists for the treatment of anaphylaxis are urgently required. In particular, intravenous preparations of modern non-sedating H1-antihistamines would be desirable.

There is little evidence for an effect of histamine H2-receptor antagonists in the treatment of acute anaphylactic reactions. One study reports a reduction in cutaneous symptoms after the additional administration of ranitidine compared with the use of a histamine H1-receptor antagonists alone in the treatment of allergic reactions [96]. There is somewhat more evidence for the prevention of hypersensitivity reactions by the addition of histamine H2-receptor antagonists, although the effect was not evaluated separately from other drugs [97, 98]. There are case reports in the literature on anaphylactic reactions caused by ranitidine [99]. The combined use of histamine H1- and H2-receptor antagonists can be attempted [100].

### Glucocorticoids

Glucocorticoids plays a secondary role in the acute phase of anaphylaxis due to their comparatively slow onset of action [101].

There are no systematic clinical studies for this indication. However, glucocorticoids are effective in the treatment of asthma. A non-specific membrane-stabilizing effect within 10–30 min of administration of very high doses of glucocorticoids (in adults, 500–1000 mg independent of the potency of the substance) has been postulated in review articles [4, 101, 102]. In the absence of intravenous access, glucocorticoids may also be given orally in syrup form or rectally in suppository form, especially in small children (e.g., prednisolone suppositories or rectal enemas) at a dose of 2 mg/kg.

In the case of slow response and unclear evidence, treatment with glucocorticoids should only be performed once vital functions have been stabilized and immediate life-saving measures have been performed, such as oxygen administration, intramuscular adrenaline use, or volume substitution!

## General aspects and treatment measures

### When and how should allergen contact be stopped?

In the case of anaphylaxis, one should first establish whether it is possible to stop further allergen exposure. In particular situations (e.g., infusions), this can be readily achieved and should be done immediately. The use of a tourniquet on an extremity and/or subcutaneous injection of adrenaline around a local allergen depot (e.g., wasp sting or injection site of allergen-specific immunotherapy) is no longer recommended, since the therapeutic benefit is questionable and there is a risk of distracting from more important measures.

### Should one call for help?

If possible, further help should be called for in order to achieve the conditions for adequate medical care. All practice-based physicians should keep emergency equipment available for the treatment of anaphylactic reactions (Table 4). The team should be regularly trained, with the option of designating tasks. In the case of severe anaphylactic reactions, the emergency services/paramedics should be alerted (in Germany, call 112).

**Table 4:** Emergency equipment for the treatment of anaphylactic reactions in the medical office

Stethoscope
Blood pressure monitor
Pulse oximeter, possibly also blood glucose meter
Tourniquet, venous catheters (in different sizes), syringes, infusion set, adhesive tape for catheter fixation
Oxygen and nebulizer set with oxygen mask (different sizes)
Bag valve mask (different sizes)
Suction device
Guedel tube where appropriate
Volume for infusion (e.g., balanced electrolyte solution)
Drugs for injection: adrenaline, glucocorticoid, histamine H1-receptor antagonist
Short-acting beta2-adrenoceptor agonist, e.g., salbutamol for inhalation (preferably as an inhalation solution for administration via a nebulizer set with mask, if necessary in metered dose with, e.g., inhalation aid/spacer/mask, autohaler)
Automated external defibrillator

### How should symptoms and complaints be recorded?

First of all, a short history should be taken and a basic physical examination carried out. This comprises the following steps summarized in a “five-second round” (Table 5) (www.aelrd.de).

**Table 5:** Five-second round for rapid evaluation of vital parameters (from A. Bohn, *Bundesverband Ärztliche Leiter Rettungsdienst Deutschland* (ÄLRD) (www.aelrd.de))

Five-second round	Examination of vital signs (spontaneous movement)
A—Airway	Muffled speech, swollen tongue
B—Breathing	Evaluation of breathing (dyspnea, stridor, wheezing; optional: auscultation, pulse oximetry)
C—Circulation	Evaluation of recap time (preferably forehead or sternum) Pulse (strength, frequency, regularity) and blood pressure
D—Disability	Consciousness, blood glucose measurement
E—Exposure	Inspection of easily visible areas of skin areas and mucous membranes, question patient regarding other symptoms (e.g., nausea, vomiting, headache, feeling of chest pressure, impaired vision, pruritus)
Secondary survey	AMPLE approach Known *A*llergies, possible elicitors of acute reaction, risk factors (asthma, other pre-existing diseases) *M*edication *P*atient history *L*ast meal *E*vents

Alarm values for vital parameters are listed in Table 6. These examinations need to be repeated over the course of treatment at regular intervals.

**Table 6:** Alarm limit values for vital signs^a^

Alarm limit values depending on age	Under 1 year	1–5 Years	6–14 Years	>14 Years
Heart rate (/min)	>160	>130	>120	>110
Blood pressure (systolic, mm Hg)	<50	<60	<60	<70
Respiratory rate (/min)	>40	>35	>30	>25
Oxygen saturation (%)	<92	<92	<92	<92

^a^These values may vary due to high individual variability and can be regarded as guide values. Evidence-based data from clinical studies are not available

Young children can be examined while held by a parent. The initial aim is to calm the child and the parents in order to create an adequate examination and treatment environment. When small children are restless, it may be difficult or impossible to examine the oral cavity and perform lung auscultation. Causing irritation with a tongue depressor may increase airway obstruction and should be avoided. In this case, and in addition to general signs of dyspnea, such as retraction of the thorax or the nasal wings, attention should be paid to other clinical signs of upper respiratory tract obstruction, such as inspiratory stridor or salivation, as well as lower airway obstruction with a prolonged expiratory phase and expiratory stridor or wheezing.

### How should severity be assessed?

**Fig. 3:**
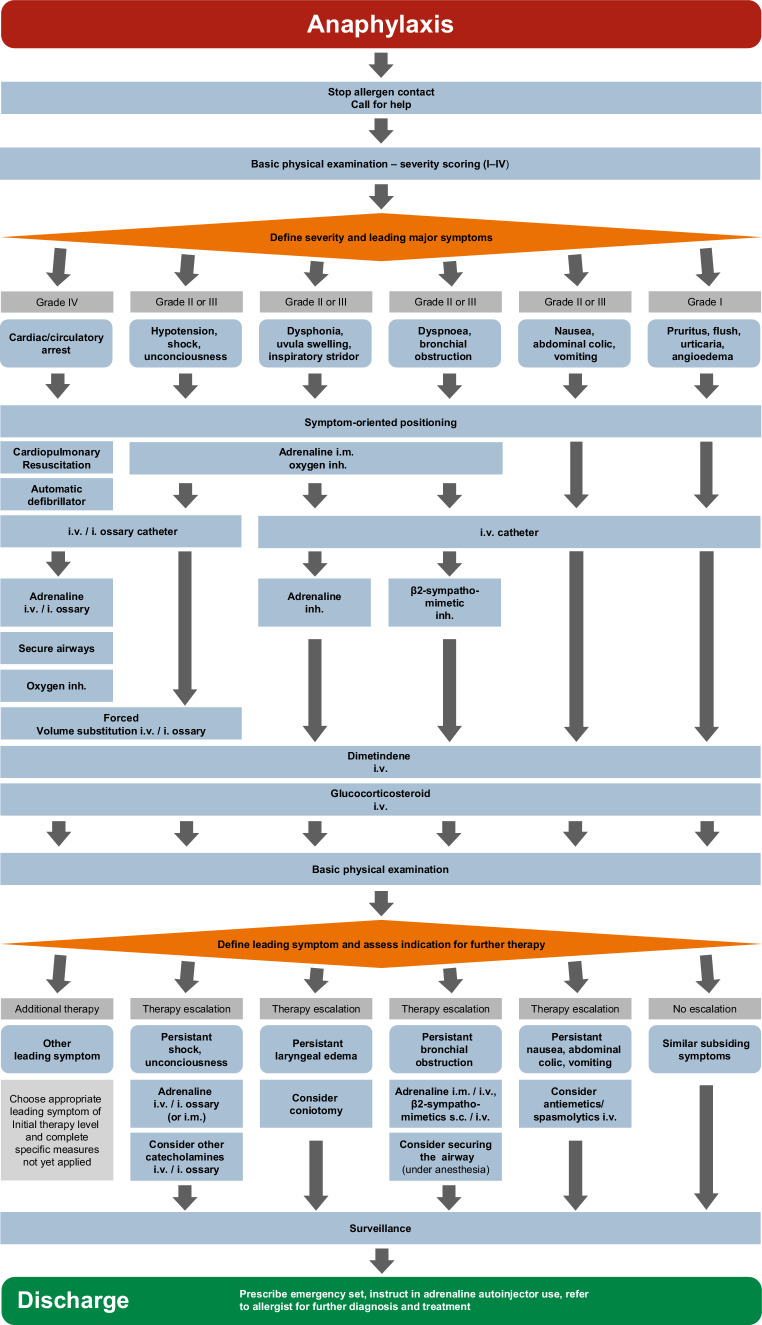
Acute treatment of the most common anaphylactic patterns. *Inh* inhaled

Based on this examination, the degree of severity of anaphylaxis should be evaluated and the most life-threatening leading symptom identified. The most life-threatening symptom needs to be treated first. This leads to the six most common scenarios ([103]; Fig. 3):

1. Anaphylaxis with cardiovascular and/or respiratory failure (grade IV anaphylaxis)
2. Anaphylaxis with a predominantly cardiovascular reaction (grade II/III anaphylaxis)
3. Anaphylaxis with predominant upper airways obstruction (grade II/III anaphylaxis)
4. Anaphylaxis with predominant lower airways obstruction (grade II/III anaphylaxis)
5. Anaphylaxis with predominant gastrointestinal involvement (grade II anaphylaxis)
6. Anaphylaxis with systemically mediated generalized skin manifestation and subjective symptoms (grade I anaphylaxis).

### How should the patient be positioned?

Immediately after examination, symptom-oriented positioning of the patient needs to be carried out. A flat position and avoidance of abrupt changes in position (sitting up, standing up) or further physical exertion (running) are a fundamental strategy. Positioning can vary according to the situation. Sitting/standing up and physical exercise (running) should be avoided due to abrupt volume shift (“venous collapse”) or aggravation of anaphylaxis (co-factors). In the case of impaired consciousness but intact circulation, especially in preclinical situations, stable side-positioning is recommended. To improve the hemodynamic situation, Trendelenburg positioning (legs up) can be performed. In situations where respiratory distress is the leading symptom (dyspnea), a half-sitting position may be better. When treating children, care must be taken not to exert any force during positioning that may increase the child’s anxiety.

### How should anaphylaxis with cardiovascular arrest be treated?

Cardiopulmonary resuscitation with chest compressions and mouth-to-mouth breathing at a ratio of 30:2 (compressions:breaths) in adults should be initiated. In children, resuscitation is initiated in line with the current European Resuscitation Council (ERC) guidelines, i.e., after five initial breaths, two breaths are given after every 15 chest compressions. An automated defibrillator should be used and early defibrillation performed in the case of ventricular fibrillation. An intravenous or intraosseous catheter is required for further drug treatment. Adrenaline (intravenous or intraosseous) at a dose of 1 mg in adults or 0.01 mg/kg is the drug of first choice and is repeated in at 3‑ to 5‑min intervals until stabilization of spontaneous circulation has been achieved [80, 81]. For sufficient oxygenation in emergency care, bag-valve-mask ventilation with 100% oxygen is sufficient. If optimization measures (positioning of the head, Guedel tube, two-person technique) are unsuccessful in the case of difficulties with mask ventilation, supraglottic airway devices are used. Laryngeal masks and laryngeal tubes can be used in all age groups. Alternatively, a pharyngeal tube can be used in small children; here, ventilation is induced via a nasal tube (tube length = tip of the nose–ear tragus) while holding the mouth and other nostril closed. Endotracheal intubation represents the final method of airway management. This can also be performed as a first step in the case of sufficient expertise. It has been shown for all age groups that endotracheal intubation should only be performed by experienced individuals [58, 104].

For successful resuscitation, it is important to compensate for the underlying volume deficiency by means of forced volume replacement, according to the pathophysiology of anaphylaxis. Immediate transfer to and treatment on an intensive care unit are recommended (Table 7).

**Table 7:** Pharmacotherapy for children, adolescents, and adults under intensive care conditions

Substance	Indication	Route of administration	Dose	<15 kg BW	15–30 kg BW	>30–60 kg BW	>60 kg BW in adults
Adrenaline 1:10,000^a^ (1 mg/10 ml)	Cardiac arrest/resuscitation	i.v./i.o.	10 µg/kg	0.1 ml/kg BW	0.1 ml/kg BW	0.1 ml/kg BW	1 mg
Adrenaline 1:1000^b^ (1 mg/ml)	Respiratory symptoms Shock	Intramuscular	10 µg/kg	0.05–0.1 ml	0.15–0.3 ml	0.3–0.6 ml	0.3–0.6 mg
Adrenaline 1:10,000^a^ (1 mg/10 ml)	In severe shock (if i.m. not possible)	Titrating i.v./i.o.	1 µg/kg	0.01 ml/kg BW	0.01 ml/kg BM	0.01 ml/kg BW	0.1–0.6 mg
Adrenaline	–	Continuous infusion	–	0.05–1.0 µg/kg/min	0.05–1.0 µg/kg/min	0.05–1.0 µg/kg/min	0.05–1.0 µg/kg/min
Adrenaline 1:1000 (1 mg/ml)	–	Inhaled via nebulizer	–	3 ml^b^	4 ml^b^	5 ml^b^	5 ml^b^
Dimetindene	–	Intravenous	0.1 mg/kg	1 ml^c^	2–3 ml^c^	4 ml^c^	8 ml^c^ or 1 ml/10 kg BW
Prednisolone	–	Intravenous	2 mg/ml	25 mg	50 mg	100 mg	250–1000 mg
Salbutamol Terbutaline	–	Inhaled	–	4–8 Puffs MDI per spacer	4–8 Puffs MDI per spacer	4–8 Puffs MDI per spacer	2–4 Puffs MDI per spacer
Reproterol^d^	–	Continuous infusion	–	0.1 µg/kg/min	0.1 µg/kg/min	0.1 µg/kg/min	0.1 µg/kg/min
Volume	–	Infusion (balanced electrolyte solution, Ringer’s acetate solution)	10–20 ml/kg	10–20 ml/kg	10–20 ml/kg	10–20 ml/kg	500–1000 ml
Oxygen	–	Nasal cannula Non-rebreather mask	–	2–12 l/min	2–12 l/min	2–12 l/min	2–12 l/min

*MDI* metered-dose inhaler, *Amp* ampoule, *BW* body weight

^a^For intravenous/intraosseous administration, 1 ml of 1:1000 solution (= 1 mg adrenaline in 1 ml of commercial solution) with 9 ml NaCl 0.9% (final concentration 1:10,000 = 0.1 mg/ml) or prefilled adrenaline syringe (1 mg/10 ml) are used

^b^For intramuscular administration and inhalation, the undiluted commercially available solution is used (adrenaline 1:1000, 1 mg/ml)

^c^Of a basic concentration for 1 mg/ml (1 ml contains 1 mg dimetindene maleate)

^d^Reproterol can also be given as a bolus

### How should anaphylaxis with a cardiovascular reaction as the leading symptom be treated?

As an immediate measure, intramuscular (body weight-adjusted) injection of adrenaline is recommended, especially when there is no intravenous catheter (Fig. 3; Table 7). In this situation, the adrenaline auto-injectors used for layperson administration may be advantageous for their rapid usability. Standardized doses of auto-injectors of 0.15 mg, 0.3 mg, or 0.5 mg are practical single doses for administration. In the case of insufficient response, the intramuscular injection can be repeated after 5–10 min.

Oxygen administration is recommended with the aim of increasing the inspired oxygen fraction (FiO_2_) to >0.5. This is possible with a non-rebreather oxygen mask. Nasal tubes do not increase FiO_2_ sufficiently.

In all cases of impaired consciousness, vomiting should be expected at any time. This needs to be considered when positioning the patient. The mouth should be opened using the Esmarch (jaw thrust) maneuver and inspected for vomited material or foreign bodies (e.g., dental prostheses). An operational suction unit is helpful.

An intravenous catheter is necessary for further treatment (Table 7). If this is not possible, an intraosseous catheter is indicated. The central aim of treatment is to compensate for the relative volume loss. Forced volume replacement with a crystalloid solution (balanced electrolyte solution) is required in the form of a volume bolus over 5 min. In adults, 500–1000 ml are administered, while in children the volume bolus is initially 20 ml/kg. A flow rate of this magnitude requires a large-lumen indwelling venous catheter (≥18 gauge) or several catheters.

In persistent or life-threatening shock, fractionated intravenous/intraosseous or intramuscular administration of adrenaline or as a continuous drip is indicated. Antiallergic drugs like histamine H1-receptor antagonists (note: antimuscarinic side-effects of sedating anthistamines!) or glucocorticoids should be used after stabilization of vital functions and administration of i.m. adrenaline (Table 7). Continuous blood pressure and pulse monitoring is indicated in these situations. With adequate expertise, other sympathomimetic drugs such as noradrenaline may be used or a continuous infusion initiated with pumps under continuous monitoring.

### How should anaphylaxis with upper airway obstruction as the leading symptom be treated?

Clinically detectable swelling in the area of the upper airways is characteristic for this situation. This may be identifiable as swelling of the tongue or uvula, dysphonia, or inspiratory stridor. These situations can become life-threatening if the laryngeal entrance is obstructed. As an immediate measure, intramuscular injection of adrenaline and oxygen administration are recommended (Fig. 3). Additional inhalation of adrenaline is also indicated in such situations (Tables 7 and 8). If the treatment response is insufficient, airway management according to the algorithm in the S1 guideline for prehospital airway management should be performed (Fig. 2; [58]).

**Table 8:** Pharmacotherapy for children, adolescents, and adults under non-intensive conditions (e.g., outpatient setting)

Substance	Administration	<7.5 kg BW	7.5–25 (–30)^d^ kg BW	30–60 kg BW	>60 kg BW
Adrenaline	Intramuscular	50–600 μg			
Adrenaline	Auto-injector i.m.	Not approved	150 µg	300 µg	1–2 × 300 µg or 500 µg
Adrenaline	Inhalation nebulizer	2–5 ml^b^			
Adrenaline	Intravenous^a^	Titrating bolus doses 1 μg/kg BW			
Dimetindene	Intravenous	1 ml^c^	1 ml/10 kg BW^c^ (max. 4 ml)	1 Amp = 4 ml^c^	1–2 Amp = 4–8 ml^c^ (1 ml/10 kg BW)
Prednisolone	Intravenous	50 mg	100 mg	250 mg	500–1000 mg
Salbutamol Terbutaline	Inhaled	2 Puffs via spacer	2 Puffs via spacer	2–4 Puffs via spacer	2–4 Puffs via spacer
Volume	Bolus (NaCl 0.9%)	20 ml/kg BW	20 ml/kg BW	10–20 ml/kg BW	10–20 ml/kg BW
Oxygen	Inhaled	2–10 l/min	5–12 l/min	5–12 l/min	5–12 l/min

*AMP* ampoule, *BW* body weight

^a^For intravenous administration, a 1-mg/ml adrenaline solution is diluted in 100 ml NaCl 0.9% (final concentration, 10 mg/ml)

^b^For inhalation, the original concentration of the commercial solution is used (1 mg/ml)

^c^An original concentration of 1 mg/ml (1 ml contains 1 mg dimetindene maleate)

^d^Various weight-dependent approvals for different auto-injectors

### How should anaphylaxis with bronchial obstruction as the leading symptom be treated?

This symptom is one of the most common in anaphylaxis. In all potentially life-threatening situations, adrenaline should be given intramuscularly. Topical bronchodilator therapy is of central importance ([105]; Fig. 3). Various short-acting beta-adrenoceptor agonists (e.g., salbutamol, terbutaline) are approved for the treatment of bronchial obstruction (Tables 7 and 8). It is important to note that patients with anaphylaxis often have little experience with inhalation therapy and can more easily use spacers for metered dose inhalers or procedures with continuous aerosol administration (such as masks for pressure/oxygen connection and electric nebulizers). This also holds true for young children and children without experience with inhalation therapy. Compact battery-driven nebulizers are now available and can also be used in emergency preclinical situations. If therapy needs to be escalated, repeated i.m. adrenaline is given. If resuscitation is imminent, intravenous administration of adrenaline can be considered. A further treatment modality is the application of an injectable beta2-adrenoceptor agonist (e.g., terbutaline s.c. or reproterole i.v.) (Table 7).

In acute severe asthma with muscular exhaustion and failure of non-invasive ventilation, emergency anesthesia with invasive ventilation may be necessary [65]. Here, the current guidelines and recommendations on anesthesia with esketamine and midazolam should be followed [104].

### How should anaphylaxis with predominantly abdominal symptoms be treated?

Anaphylaxis with predominantly abdominal symptoms is treated in the same way as anaphylaxis with generalized skin symptoms (Fig. 3). Only if there is insufficient response to systemically administered antiallergic drugs will gastrointestinal symptoms be treated separately. Nausea, vomiting, or abdominal cramps may be the relevant symptoms. Antiemetics such as metoclopramide, antihistamines (e.g., dimenhydrinate) or serotonin-[5-HT3] antagonists (e.g., ondansetron) can be used for treatment. For abdominal cramps, intravenous administration of a muscarinic receptor antagonist (butylscopolamine) may be considered.

### How should anaphylaxis with predominantly skin symptoms be treated?

Placing an intravenous catheter is the first measure taken. A crystalloid solution drip (e.g., balanced electrolyte solution) is recommended to keep this open. Antiallergic drugs such as dimetindene or glucocorticoids are applied at usual doses (Fig. 3; Table 8).

## Special aspects of hospital treatment

### Which emergency drugs should be stored in the emergency department or on the ward?

In emergency departments and on wards where provocation tests or allergy procedures with increased risk for anaphylaxis are performed, up to two adrenaline auto-injectors each in the doses 300 μg or 500 μg should be kept available. If the treatment of children is expected, a further two 150-µg adrenaline auto-injectors must be kept available. Adrenaline inhalation using a nebulizer should be available. In addition, injectable histamine H1-receptor antagonists and glucocorticoids, as well as salbutamol for inhalation with appropriate devices (spacer or moist inhalation), should be on hand.

### How should patients with acute anaphylaxis be treated in the emergency department?

Individuals presenting to the emergency department with suspected anaphylaxis need to be treated immediately. In order to make the diagnosis of “anaphylaxis,” the clinical criteria need to be applied (see above). In addition to an immediate evaluation of clinical signs and symptoms (Fig. 3), continuous monitoring of circulation, including measurement of pulse, blood pressure, and peripheral oxygen via pulse oximetry, must be established. Patients with severe anaphylactic reactions (e.g., requiring an adrenaline auto-injector) should be hospitalized and supervised for 24 h due to the risk of a biphasic (bimodal) reaction.

### How should anaphylaxis be treated on the ward?

Elicitors of anaphylaxis on the ward are often drugs used there for treatment. Reactions to parenterally administered substances occur rapidly after use. In the case of substances for enteral administration, delayed symptoms are also possible. The first measure to be taken is to discontinue allergen exposure and—depending on the grade of severity—alert the emergency team.

### What are the special considerations for planned provocation testing in anaphylaxis?

Whenever a procedure that carries a risk of anaphylaxis is planned (allergy provocation test, allergen-specific immunotherapy with hymenoptera venom), careful preparation is essential, including:

- Monitoring sheet with emergency medication and emergency plan.
- Emergency drugs prepared in weight-adjusted doses near to the patient.
- Rapid medical care if allergic symptoms occur.
- Intravenous catheters for rapid administration of intravenous drugs and volume.
- The indication to administer medication is established according to the flow diagram (Fig. 3).

It is advisable that affected patients or, in the case of young children, their parents learn to use the auto-injector themselves under close instruction from medical personnel in order to train for administration at home. If possible, training should be with the same type of auto-injector as the one that will be used by the patient. This approach enables patients to gain confidence in administering an auto-injector and reduce the fear associated with its use.

### How should anaphylaxis be treated on the intensive care unit?

One advantage of high care (on an intensive care unit) with continuous monitoring is that shock states can be detected and treated earlier. If the intensive care personnel become aware of anaphylaxis, whether due to hypotension, tachycardia, a warning signal from the monitor, or low oxygen saturation, the principal procedure does not differ from other settings. Common elicitors of anaphylaxis on an intensive care unit include drugs and blood products; therefore, the very first measure to take is to discontinue administration of the potential allergen or elicitor. The priority-oriented ABCDE (airway, breathing, circulation, disability, and exposure) approach (Table 5) described above is then initiated. Depending on the ongoing mode of intravenous catecholamine treatment, syringe pumps may need to be adapted. Adrenaline should be given due to its mast-cell stabilizing action, which is unique among catecholamines. One needs to bear in mind that, under continuous vasopressor treatment, the normal intramuscular administration of 0.3–0.5 mg adrenaline may have reduced efficacy due to peripheral hypoperfusion. Therefore, intravenous administration in intensive care is performed in 50 μg bolus doses in adults and 1 μg/kg bolus doses in children [81] until the patient is stabilized, preferably via a central venous catheter; alternatively, peripheral administration is also possible.

### What needs to be considered in discharge management after an anaphylactic reaction?

Following successful treatment of anaphylaxis, patients, and/or their relatives where appropriate, should be informed about the condition and undergo adequate allergy diagnostics (Table 8). If anaphylaxis occurred intraoperatively, an anesthesia document needs to be issued and the patients must be informed about the reaction. It is absolutely essential to document reactions, together with symptoms, co-factors, and possible elicitors. An emergency first-aid kit is prescribed (see below).

Patients with food allergies should receive an individually tailored therapeutic elimination diet under the guidance of a nutritionist with allergy expertise (in Germany, the addresses of certified specialists can be obtained from the German Allergy and Asthma Association [*Deutscher Allergie- und Asthmabund*, DAAB] and the working group on dietetics in allergology [*Arbeitskreis Diätetik in der Allergologie*]). Following reactions to insect stings, the possibility of allergen-specific immunotherapy should be discussed.

If the elicitor of anaphylaxis with extracutaneous symptoms cannot be reliably avoided (e.g., insect stings, foods) or there is an increased anaphylaxis risk, patients should be advised to carry an emergency first-aid kit with them at all times, together with a written document, e.g., anaphylaxis passport (see section below on patient management and self-medication). The patient should receive instructions regarding emergency management and the use of emergency medication. If the elicitor is a drug used in the hospital setting, an allergy passport should be provided with detailed documentation of the reaction in order to allow allergy diagnostics. In the case of recurrent reactions, an attempt at long-term pharmacological treatment can be considered, such as long-term administration of antihistamines or an anti-IgE antibody such as omalizumab [106, 107].

For questions regarding everyday management, particularly in food-induced anaphylaxis, patients should be referred to a patient organization for support (e.g., in Germany, the DAAB).

### How should perioperative anaphylaxis be managed?

During analgosedation or general anesthesia, the patient is not able to communicate early symptoms such as itch or nausea; therefore, continuous supervision and observation of respiratory and cardiovascular function is important. If unexpected hypotension or tachycardia occur in the perioperative setting, other possible symptoms of anaphylaxis need to be immediately sought:

- Is erythema, swelling, or urticaria developing, possibly beginning on the arm receiving the infusion?
- Is there prolonged expiration with reduced expiratory flow or a decrease in pulse oximetry oxygen saturation?
- Is there decreased lung compliance?

The differential diagnosis in the context of viscerosurgical procedures needs to differentiate eventeration syndrome, which may manifest clinically as prostacyclin-mediated flushing, tachycardia, and hypotension.

If the working diagnosis severe anaphylaxis or anaphylactic shock is confirmed, treatment with adrenaline is immediately initiated. In adults with severe shock but maintained circulation, titrated adrenaline in 0.1- to 0.3-mg bolus doses is administered until systolic blood pressure rises to 100 mm Hg. At the same time, volume therapy is initiated with 1–3 l of balanced electrolyte solution. Histamine H1-receptor antagonists and optionally H2-antagonists and glucocorticoids, as described above, are then administered intravenously. Extended hemodynamic monitoring should be considered. If these measures are able to stabilize the patient, one needs to decide whether and to what extent the surgical procedure can be performed or continued. Further intensive monitoring is recommended depending on severity.

## What are the special aspects of treatment in the medical office?

### Which elicitors of anaphylaxis are common in the medical office?

Possible elicitors in the medical office include allergen solutions used in allergen-specific immunotherapy (hyposensitization), as well as natural rubber latex, local anesthetics, and drugs used in the medical office (e.g., antibiotics, cyclooxygenase inhibitors, radiographic contrast media, vaccines, and intravenous iron). Furthermore, patients with severe allergic reactions to hymenoptera venoms or foods may present to the nearest medical office.

### How should a medical office prepare for emergency treatment?

All medical offices should keep emergency equipment available for the treatment of anaphylaxis. Since anaphylactic reactions are not a regular occurrence in most medical offices, regular training in anaphylaxis recognition, as well as pharmacological and non-pharmacological treatment, needs to be performed (especially with regard to distribution of tasks, positioning, calling for help, oxygen, recording respiratory and cardiovascular function). Regular training of team procedures during anaphylaxis improves medical care in the emergency situation. A written, easily accessible emergency plan with a description of the necessary drugs and dosages is highly recommended. Patients should be treated in a room separated from other patients yet easily accessible to several caregivers. In the pediatric setting, the weight-adjusted dosages for emergency therapy in children with allergen-specific immunotherapy can already be noted on the documentation sheet.

It is helpful to define the following specifications in preparation for an emergency situation:

- Where is the emergency equipment stored?
- What is the exact procedure in an emergency?
- Who is responsible for what? (inform the physician, take care of the patient, call for help, etc.)
- In which room will the patient be treated?

### What emergency equipment should a medical office keep available?

The elements shown in Table 4 are recommended as emergency equipment in a medical office. A pulse oximeter should be available, whereas ECG and blood pressure monitors are not standard equipment in all medical offices.

### What is the procedure in an emergency?

Acute treatment is performed as described above (treatment in the hospital) with no essential differences.

Adrenaline is also administered in the medical office by physicians not experienced in emergency treatment, preferably intramuscularly. Administration can be repeated if circulation is not stabilized. If there is no monitor, heart rate and blood pressure can be measured, while the capillary refill time on the skin of the sternum or fingertip can provide information on cardiovascular function.

Caution should be taken with the intravenous administration of adrenaline; this should be reserved for physicians with expertise and requires continuous blood pressure and pulse monitoring (exception: resuscitation situation with an i.v. catheter already in place).

In the case of anaphylaxis with medium to severe involvement of the respiratory or cardiovascular system, the emergency medical and rescue services should be promptly alerted following discontinuation of allergen exposure (in Germany, call 112).

### How is discharge managed in the medical office?

Since the possibilities for patient monitoring are limited in the medical office, transfer to a hospital for monitoring is recommended in the case of severe or not reliably classifiable anaphylaxis. Otherwise, discharge management is the same as from hospital, as listed above (Table 9).

**Table 9:** Important aspects of discharge management of anaphylaxis patients

*Identification of the elicitor*	Allergy history
	If necessary, referral to allergy diagnostics (specific IgE and/or skin test)
*Recommendations for the prevention of renewed reactions*	In food allergy: individually tailored therapeutic elimination diet (patient organizations, e.g., DAAB, “dietetics in allergy” working group, German Society for Nutritional Medicine)
	In insect venom allergy: consider indication for allergen-specific immunotherapy
	In drug allergy: avoidance and allergy passport recommended
*Recommendations for pharmacological self-management*	Written plan for pharmacological self-management (anaphylaxis passport)
	Prescription of emergency drugs in body weight-adjusted doses
	Training in administration
*Recommendations for everyday management*	Information on support from patient organizations for problems in childcare facilities, school, shopping, travelling
	Information regarding non-labeled allergens in foods
	Information on and referral to specialized allergy-trained nutritionists

*DAAB* German Allergy and Asthma Association (*Deutscher Allergie- und Asthmabund*)

## Special aspects in childhood

With regard to the dosing of certain drugs used in the treatment of anaphylaxis, the special dosages for children need to be taken into account.

## Patient management and self-medication

### Target groups for the prescription of an emergency first-aid kit, including an adrenaline auto-injector

Indications for the prescription of an emergency first-aid kit, including an adrenaline auto-injector, are listed in Tables 10 and 11.

**Table 10:** Indications for the prescription of an adrenaline auto-injector

Patients with systemic allergic reactions and bronchial asthma (even with no history of anaphylaxis)
Progressive severity of symptoms of the systemic allergic reaction
History of prior anaphylactic reactions to elicitors that cannot be reliably avoided
Systemic allergy with extracutaneous symptoms to potent allergens such as peanuts, tree nuts, milk, sesame
Strong sensitization with increased risk of anaphylaxis—prior to allergy provocation testing
Patients that react to minute amounts of allergen
Adults with mastocytosis (also without known anaphylaxis)

**Table 11:** Indications for the prescription of an additional (second) adrenaline auto-injector

History of extremely severe anaphylaxis
Obesity >100 kg BW
Uncontrolled bronchial asthma
Nearest emergency medical care is poorly accessible
Especially high risk for severe anaphylaxis (e.g., adults with mastocytosis after anaphylaxis)
Organizational: second auto-injector for childcare facility, school, or depending on the family situation

We recommend that all patients with:

1. A history of anaphylaxis
2. Systemic allergic reactions with extracutaneous symptoms and high future risk of anaphylaxis (e.g., due to well-known potent allergens such as peanut, tree nuts, milk, sesame) or at high risk for life-threatening reactions (e.g., bronchial asthma)
3. Highly sensitized persons without previous anaphylactic reactions but high-grade suspicion of an increased anaphylaxis risk—prior to allergy provocation testing

be provided with an emergency first-aid kit if the elicitor cannot be reliably avoided, as in the case of, e.g., insect venom or food anaphylaxis. The precise indications are given in more detail in Table 10. It is normally possible to avoid an elicitor in the case of drug-induced anaphylaxis following adequate allergy diagnostics, education, and the issuing of an allergy passport.

### Can an emergency first-aid kit also be prescribed in patients with high-grade suspicion for anaphylaxis prior to allergy testing?

Children with atopic eczema (atopic dermatitis, eczema) are often sensitized to common food allergens, especially after early onset severe eczema [108, 109]. In the case of clinically relevant sensitizations, these patients often experience an anaphylactic reaction after first oral exposure. In order to evaluate the clinical relevance of this, an oral food challenge is performed, often in the inpatient setting [110]. Since there is often an interval of several weeks or months between the time of indication and the performance of oral challenge tests, patients can be given an adrenaline auto-injector in the interim. Instruction for use with the help of an auto-injector trainer (“dummy”) is essential. When considering the rationale for this approach, one needs to consider the time interval until oral challenge testing, the likelihood of a clinically relevant food allergy, and the probable severity of a reaction depending on concomitant diseases such as asthma, as well as the likelihood of accidental exposure.

In peanut or tree nut allergy, the risk of a systemic reaction increases with the concentration of allergen-specific IgE antibodies against storage proteins [109, 111]. The best predictor of clinical relevance is specific IgE against the 2S albumins (Ara h 2 in peanut, Cor a 14 in hazelnut, and Ana o 3 in cashew). In some patients, 2S albumin-specific IgE is so high that one can assume with 90–95% probability that these patients will suffer an anaphylactic reaction even though they have never have eaten this food allergen before [109, 111]. Oral food challenges are often not performed in these patients, or only at a later point in time (e.g., at the time of school entrance). These patients can be diagnosed with “high-grade suspicion” of, e.g., peanut or tree nut allergy without ever having experienced a clinical reaction. These patients should also receive an adrenaline auto-injector, as well as therapeutic nutrition counselling in order to consistently avoid the highly suspected foods and, where necessary, replace nutrients lacking in their avoidance diet.

### What should the emergency first-aid kit contain?

In German-speaking countries, physicians often prescribe several drugs for patients to be put together in an emergency first-aid kit, which should be carried at all times together with the anaphylaxis passport. The authors recommend an adrenaline auto-injector, a histamine H1-receptor antagonist, a glucocorticoid, and, in patients with bronchial asthma or a prior reaction with bronchospasm, an inhaled bronchodilator (beta2-adrenoceptor agonist) (Table 12).

**Table 12:** Contents of an emergency first-aid kit for patients with anaphylaxis

Adrenaline	Auto-injector for intramuscular, body weight-adjusted administration	
	>7.5 to 25 kg BW or >15–30 kg BW:	150 μg^a^
	>25 to 50 kg BW or >30–50 kg BW:	300 μg^a^
	>50 kg BW:	300–500–600 μg
Histamine H1-receptor antagonist	Depending on patient age and preference, orally as fluid or (lozenge) tablet. The dose of the non-sedating antihistamine may be increased up to four times a single dose. For dimetindene drops, weight-adjusted dose, as with the intravenous formulation, can also be recommended as an oral dose (see Table 8)	
Glucocorticoid	According to patient age and preference, oral (liquid or tablet) or rectal with 50–100 mg prednisolone equivalent	

*BW* body weight

In known bronchial asthma or previous reaction with bronchospasm: additionally a beta2-adrenoceptor agonist

If severe obstruction of the upper airways is expected (laryngeal edema), additionally an inhaled adrenaline preparation with spray head for pharmaceutical vials (needs to be specially requested from the pharmacist)

Note: An emergency first-aid kit should include an anaphylaxis passport with written instructions on how to use the contents

^a^Approval differs for each auto-injector preparation

When selecting the histamine H1-receptor antagonist, swallowing ability and individual preference in terms of form of administration should be taken into consideration (drops for small children, tablets or lozenges for older children and adults). If there is a history of difficulty in swallowing (e.g., laryngeal edema), administration in liquid form is preferred. The same criteria apply to glucocorticoids (1–2 mg/kg BW), whereby rectal administration is also possible. The expert group recommends the treatment of anaphylaxis with antihistamines in increased doses (up to four times the approved single dose). The new second-generation selective histamine H1-receptor antagonists are not approved for the treatment of anaphylaxis; however, they can be recommended alongside sedating antihistamines for oral emergency treatment, since they have shown a rapid onset of action in placebo-controlled skin test studies and fewer side effects, such as sedation. For asthma patients, inhaled beta2-adrenoceptor agonists are additionally prescribed. Alternatively, in the case of a prior history of laryngeal edema, an adrenaline preparation for inhalation may be prescribed.

There are various commercially available adrenaline auto-injectors that differ in dose, handling, injection mechanism, and needle length. Special instruction is necessary, the preparations cannot be easily substituted, and repeat prescriptions need to be organized [112]. The “aut idem” box needs to be ticked on the prescription.

### When should two adrenaline auto-injectors be prescribed?

Table 11 shows a list of indications in which two adrenaline auto-injectors should be prescribed.

The dosing of adrenaline for self-management in anaphylaxis is a less controlled initial measure and does not need to be the same as adrenaline administration under medical supervision and appropriate monitoring. Due to a lack of data, dose recommendations for self-management are given for children in approximate relation to body weight, but are not calculated directly from body weight in adults [113]. There is scant evidence on the optimal adrenaline dose or number of adrenaline auto-injectors required for self-management. There are cases in which physicians or patients feel it necessary to prescribe a second adrenaline auto-injector. Co-existing bronchial asthma is a well-known risk factor for use of a second adrenaline injector. The authors recommend prescribing a second adrenaline auto-injector for the indications listed in Table 11.

It is important to ensure that patients are prescribed a second auto-injector, or replacement auto-injector, that uses the same technique as their previous device. Patients should carry their emergency first aid kit for immediate first aid with them at all times. From an organizational perspective, it makes sense in individual cases to prescribe two adrenaline auto-injectors for different locations (e.g., school, childcare facility, workplace, and in the case of separated parents). However, this can lead to confusion and lack of protection compared to patients that have their auto-injector on them at all times. Therefore, the group recommends the prescription of a single auto-injector to be carried at all times.

### When is an adrenaline auto-injector no longer indicated?

An expert group of the European Academy of Allergy and Clinical Immunology (EAACI) has intensively addressed the topic of the need for patients with insect venom allergy to carry emergency self-medication at all times [114] and has formulated recommendations on the prescription of adrenaline auto-injectors, which have been included in the current guidelines for the treatment of insect venom allergy [46]. According to these recommendations, the prescription of an adrenaline auto-injector is no longer necessary when the risk of a renewed systemic reaction is approximately comparable to that of the normal population. This can be assumed after successful immunotherapy and a well-tolerated sting reaction—either after a field sting or after a sting challenge.

After completion of allergen-specific immunotherapy, adrenaline auto-injector prescription is no longer necessary in patients with only cutaneous/mucosal symptoms (grade I) or in patients that have reacted with more than cutaneous symptoms (grade II), but have no additional risk factors for non-response to venom immunotherapy [114]. Risk factors include severe insect sting reaction (grade III or IV), bee venom allergy, high risk of exposure (e.g., bee-keeper), a systemic response under immunotherapy, mast cell disease, elevated basal serum tryptase, and treatment with ACE inhibitors. Whether the auto-injector can be dispensed with once the maintenance dose of venom immunotherapy has been reached is discussed controversially. Between 70 and 80% of experts felt that patients with only cutaneous/mucosal symptoms (grade I) would not need an auto-injector once the maintenance dose had been reached. Table 10 lists indications for the prescription of adrenaline auto-injectors.

### What should training with the emergency first-aid kit include?

Most anaphylactic emergencies occur in the course of everyday life, often at home. Therefore, training on emergency self-management should cover all measures that patients need to consider or take themselves in the case of a (renewed) emergency situation. The patient should be instructed on how to:

1. Recognize anaphylactic reactions
2. Administer symptom-related self-medication
3. Store drugs correctly
4. Make an emergency call for help (in Germany and Austria, call 112, in Switzerland, 144; report anaphylaxis/anaphylactic shock, follow telephone instructions given by the rescue center)

Potential elicitors (foods, insects, drugs) should be preserved if possible.

**Fig. 4:**
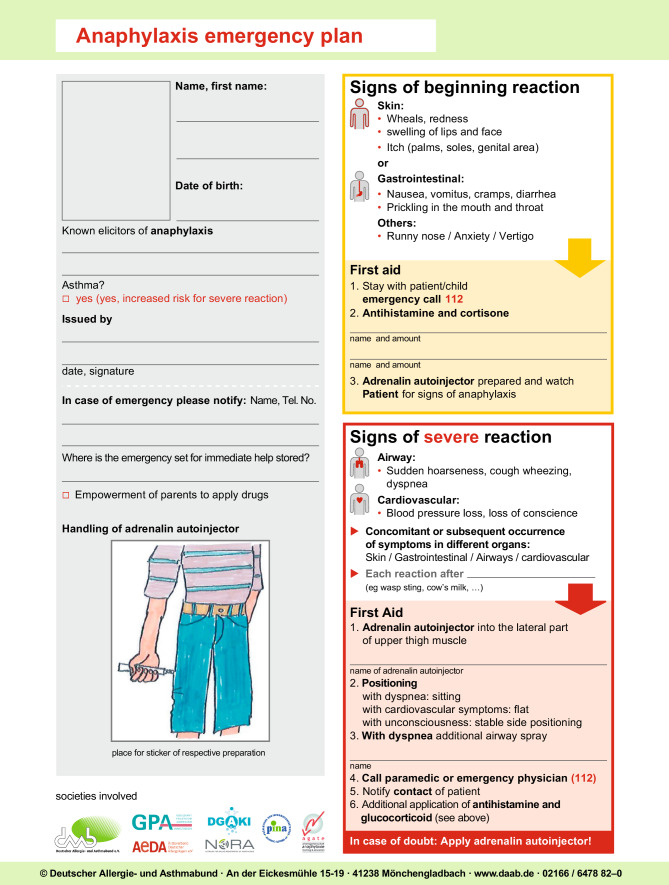
Anaphylaxis emergency plan

Self-medication is given according to symptoms and the degree of certainty that allergen contact has occurred: the correct stage-appropriate administration of a number of drugs for acute medication is an essential part of patient information, since the highest degree of uncertainty is found in this regard among patients and their families. If it is certain that contact with an anaphylaxis elicitor has taken place (insect sting without successful allergen-specific immunotherapy, eating allergy-eliciting food, use of an allergy-eliciting drug), the anaphylaxis emergency plan needs to be followed (Fig. 4). The emergency plan and anaphylaxis passport are important aids that the allergy sufferer should have on them at all times.

All patients with a history of anaphylaxis or their relatives/guardians should receive individual instruction, including a practical demonstration of how to handle the emergency kit, at the time of prescribing.

This training includes: information about the elicitors of anaphylaxis, their avoidance, referral to a nutritionist with allergy experience for advice on a therapeutic elimination diet in the case of food-induced anaphylaxis, the symptoms of anaphylaxis, as well theoretical and practical knowledge (including a demonstration) on how to administer emergency drugs in the event of a renewed anaphylactic episode. These instructions should be repeated with each new prescription of an adrenaline auto-injector. The patient, as well as those in their social environment, especially care-takers of children, also need to be instructed in the use of self-medication. Standardized anaphylaxis emergency plans are available to this end (Fig. 4), as are anaphylaxis documents and recommendations for emergency management in childcare facilities and schools. A summary of self-management instructions is also given in the anaphylaxis passport, which, in addition to triggers, includes drug dosage and mode of administration depending on the clinical symptoms.

### Who should receive anaphylaxis group education?

Anaphylaxis group education is recommended for all patients at high risk for renewed anaphylaxis. In addition to the mandatory individual instruction with the emergency first-aid kit, the German working group on anaphylaxis training and education (*Arbeitsgemeinschaft Anaphylaxie Training und Edukation*, AGATE) has developed an outpatient educational program. The program (consisting of individual educational units, 2 × 3 h), uses a target-oriented and self-contained concept comprising written, standardized, and interdisciplinary components for patients with anaphylaxis and carers of children and adolescents (e.g., parents), including a manual, documentation for participants, and sample time schedules [113, 115]. The educational program focuses on practical aspects of self-management, including role-playing, e.g., on handling adrenaline auto-injectors, as well as allergen avoidance strategies, behavior in high-risk situations in everyday life, and possibilities of risk management. It is often necessary to individualize training to address well-known individual problems and expectations. The authors recommend anaphylaxis group training whenever available. At present, anaphylaxis training programs are generally not reimbursed by insurances and hence not available in all regions of Germany.

### Where can one find further aids for the everyday management of anaphylaxis?

In addition to standardized written anaphylaxis emergency plans and the anaphylaxis passport, other information materials (such as brochures, shopping guides, restaurant maps with information on specific food allergies for the kitchen, documents for air travel, etc.) can be made available by patient organizations (in Germany, e.g., the DAAB). These organizations also provide support in everyday management (Table 9).

Adrenaline auto-injection trainers and training material are also available from the manufacturers of adrenaline auto-injectors, as well as from the DAAB. Further aids (bags for the emergency kit, as well as stickers or clothing with warning messages) can be ordered via patient organizations or the internet. SOS emergency bracelets or capsules are not yet popular in German-speaking countries and often go unnoticed in emergency situations.

It seems important to involve not only affected patients and their social environment (e.g., parents), but also physicians active in the field of allergy and anaphylaxis management, as well as other occupational groups such as paramedics, emergency services, hospital emergency departments, organizers of first aid courses, and patient organizations [116].

Overall, the acute management of anaphylaxis patients in Germany can be positively evaluated; however, there are still considerable problems in long-term management, e.g., in children in daycare centers and schools, as well as shortcomings in the prompt administration of adrenaline, further diagnostics, training, and education [28, 75].

It is important to make the medical profession aware of this potentially life-threatening problem and familiarize them with the possibility of intramuscular adrenaline administration, including the use of adrenaline auto-injectors for self-medication.

## Anaphylaxis after COVID-19 vaccination

Recently vaccination programs against COVID-19 have been started in several countries. Rare cases of severe allergic reactions have been reported from United Kingdom and USA; this has led to unsettledness and fear among patients and vaccinating physicians. The guideline group and allergological societies therefore developed position statements [117, 118] stressing the fact that some patients with defined allergic conditions may be at increased risk for anaphylaxis after COVID-19 vaccination, especially patients with severe allergic reactions to drugs or vaccines as well as known hypersensitivities to ingredients of the respective vaccines. In unclear cases allergy diagnostics should be performed prior to COVID-19 vaccination; also patients should be observed over 30 minutes after vaccine injection. Physicians and health personel involved in vaccination centers should be aware of possible risks of anaphylaxis and the necessary acute treatment modalities [117, 118].

## Addendum

### Production of the text

Following a decision of the board of the German Society for Allergy and Immunology (DGAKI) in 2017, the “anaphylaxis” working group was asked to update the text of the present guidelines together with other scientific societies and experts from other areas. In addition to allergology, these experts come from anesthesiology and intensive medicine, dermatology, pediatrics, internal medicine, pulmonology, otolaryngology, psychosomatics, pharmacology, emergency medicine, and theoretical surgery.

Allergy societies in Austria and Switzerland, as well as representatives of patient organizations, have also participated.

In addition to members of the DGAKI, representatives of the Society of practicing allergists (*Ärzteverband Deutscher Allergologen*, AeDA), Society for pediatric allergology and environmental medicine (*Gesellschaft für pädiatrische Allergologie und Umweltmedizin*, GPA), College of children and adolescents physicians in Germany (*Berufsverband der Kinder- und Jugendärzte Deutschlands*, BVKJ), Society for neonatology and pediatric intensive medicine (*Gesellschaft für Neonatologie und pädiatrische Intensivmedizin*, GNDPI), German Academy for Allergology and Environmental medicine (*Deutsche Akademie für Allergologe und Umweltmedizin*, DAAU), Austrian Society for Allergology and Immunology (*Österreichische Gesellschaft für Allergologie und Immunologie*, ÖGAI), Swiss Society for Allergology and Immunology (*Schweizerische Gesellschaft für Allergologie und Immunologie*, SGAI), German Society for Anesthesiology and Intensive Medicine (*Deutsche Gesellschaft für Anästhesiologie und Intensivmedizin*, DGAI), German Society for Pharmacology (*Deutsche Gesellschaft für Pharmakologie*, DGP), Working group anaphylaxis training and education (*Arbeitsgemeinschaft Anaphylaxie Training und Edukation*, AGATE) as well as the patient organization German Allergy and Asthma Foundation (*Deutscher Allergie- und Asthma Bund*, DAAB) have cooperated.

Consensus conferences were held in Wiesbaden in September 2017, in Mainz in March 2018, in Kassel in June 2018, in Mainz in March 2019, and in Hannover in September 2019. The recommendations elaborated during the conferences are based on a literature search evaluating clinical studies, case series, individual case reports, experimental investigations, as well as participants’ experiences and theoretical considerations. Case series were assigned the highest level of relevance, whereas theoretical considerations were only included when neither single case reports nor case series or experimental investigations were available for evaluation. The number of scientifically reliable studies on anaphylaxis is overall so limited that management remains empirical in many aspects and is often based on pathophysiological considerations.

#### Development stage

S2k

#### AWMF Guidelines register number

061-025

#### Completed

2021

#### Next revision

2025

#### ICD-10 numbers

T 78, T 80, J 45, L 23

## Abbreviations

ACE: Angiotensin converting enzyme
AGATE: German Working Group of Anaphylaxis Training and Education
CD: Cluster of differentiation
DAAB: Patient organization German Allergy and Asthma Association
EIA: Exercise-induced anaphylaxis
FDEIA: Food-dependent exercise-induced anaphylaxis
FiO_2_: Inspired oxygen fraction
ICD: International Statistical Classification of Diseases and Related Health Problems
IgE: Immunoglobulin E
i.m.: Intramuscular
i.v.: Intravenous
LT: Leukotriene
NSAID: Non-steroidal anti-inflammatory drugs
PAF: Platelet-activating factor
s.c.: Subcutaneous

## References

[1] Johansson SG, Bieber T, Dahl R, Friedmann PS, Lanier BQ, Lockey RF (2004). Revised nomenclature for allergy for global use: Report of the Nomenclature Review Committee of the World Allergy Organization. J Allergy Clin Immunol.

[2] Ring J (2010). Anaphylaxis.

[3] Simons FE, Ardusso LR, Bilò MB, El-Gamal YM, Ledford DK, Ring J (2011). World Allergy Organization anaphylaxis guidelines: summary. J Allergy Clin Immunol.

[4] Tryba M, Ahnefeld FW, Barth J, Dick W, Doenicke A, Fuchs T (1994). Akuttherapie anaphylaktoider Reaktionen. Ergebnisse einer interdisziplinären Konsensuskonferenz. Allergo J.

[5] Ring J, Brockow K, Duda D, Eschenhagen T, Fuchs T, Huttegger I (2007). Akuttherapie anaphylaktischer Reaktionen. Leitlinie der Deutschen Gesellschaft für Allergologie und klinische Immunologie (DGAKI), des Ärzteverbandes Deutscher Allergologen (ÄDA), der Gesellschaft für Pädiatrische Allergologie und Umweltmedizin (GPA) und der Deutschen Akademie für Allergologie und Umweltmedizin (DAAU). Allergo J.

[6] Ring J, Beyer K, Biedermann T, Bircher A, Duda D, Fischer J et al. Leitlinie zu Akuttherapie und Management der Anaphylaxie. S2-Leitlinie der Deutschen Gesellschaft für Allergologie und klinische Immunologie (DGAKI), des Ärzteverbands Deutscher Allergologen (AeDA), der Gesellschaft für Pädiatrische Allergologie und Umweltmedizin (GPA), der Deutschen Akademie für Allergologie und Umweltmedizin (DAAU), des Berufsverbands der Kinder- und Jugendärzte Deutschlands (BVKJ), der Österreichischen Gesellschaft für Allergologie und Immunologie (ÖGAI), der Schweizerischen Gesellschaft für Allergologie und Immunologie (SGAI), der Deutschen Gesellschaft für Anästhesiologie und Intensiv-medizin (DGAI), der Deutschen Gesellschaft für Pharmakologie (DGP), der Deutschen Gesellschaft für Psychosomatische Medizin (DGPM), der Arbeitsgemeinschaft Anaphylaxie Training und Edukation (AGATE) und der Patientenorganisation Deutscher Allergie- und Asthmabund (DAAB). Allergo J Int 2014;23:96–112 10.1007/s15007-020-4750-0 PMC7878028 33612982

[7] Muraro A, Roberts G, Clark A, Eigenmann PA, Halken S, Lack G (2007). The management of anaphylaxis in childhood: position paper of the European academy of allergology and clinical immunology. Allergy.

[8] Sampson HA, Muñoz-Furlong A, Campbell RL, Adkinson NF, Brock SA, Branum A (2006). Second symposium on the definition and management of anaphylaxis: summary report—Second National Institute of Allergy and Infectious Disease/Food Allergy and Anaphylaxis Network symposium. J Allergy Clin Immunol.

[9] Bresser H, Sander CH, Rakoski J (1995). Insektenstichnotfälle in München. Allergo J.

[10] Mehl A, Wahn U, Niggemann B (2005). Anaphylactic reactions in children—a questionnaire-based survey in Germany. Allergy.

[11] Mostmans Y, Grosber M, Blykers M, Mols P, Naeije N, Gutermuth J (2017). Adrenalin in anaphylaxis treatment and self-administration: experience from an inner city emergency department. Allergy.

[12] Moneret-Vautrin DA, Morisset M, Flabbee J, Beaudouin E, Kanny G (2005). Epidemiology of life-threatening and lethal anaphylaxis: a review. Allergy.

[13] Helbling A, Hurni T, Mueller UR, Pichler WJ (2004). Incidence of anaphylaxis with circulatory symptoms: a study over a 3-year period comprising 940,000 inhabitants of the Swiss Canton Bern. Clin Exp Allergy.

[14] Decker WW, Campbell RL, Manivannan V, Luke A, Sauver StJL, Weaver A (2008). The etiology and incidence of anaphylaxis in Rochester, Minnesota: a report from the Rochester Epidemiology Project. J Allergy Clin Immunol.

[15] Sheikh A, Hippisley-Cox J, Newton J, Fenty J (2008). Trends in national incidence, lifetime prevalence and adrenaline prescribing for anaphylaxis in England. J R Soc Med.

[16] Poulos LM, Waters AM, Correll PK, Loblay RH, Marks GB (2007). Trends in hospitalizations for anaphylaxis, angioedema, and urticaria in Australia, 1993–1994 to 2004–2005. J Allergy Clin Immunol.

[17] Worm M (2010). Epidemiology of anaphylaxis. Chem Immunol Allergy.

[18] Beyer K, Eckermann O, Hompes S, Grabenhenrich L, Worm M (2012). Anaphylaxis in an emergency setting—elicitors, therapy and incidence of severe allergic reactions. Allergy.

[19] Turner PJ, Gowland MH, Sharma V, Ierodiakonou D, Harper N, Garcez T (2015). Increase in anaphylaxis-related hospitalizations but no increase in fatalities: an analysis of United Kingdom national anaphylaxis data, 1992–2012. J Allergy Clin Immunol.

[20] Mullins RJ, Wainstein BK, Barnes EH, Liew WK, Campbell DE (2016). Increases in anaphylaxis fatalities in Australia from 1997 to 2013. Clin Exp Allergy.

[21] Lee S, Hess EP, Lohse C, Gilani W, Chamberlain AM, Campbell RL (2017). Trends, characteristics and incidence of anaphylaxis in 2001–2010: a population-based study. J Allergy Clin Immunol.

[22] Jakob R (2018). ICD-11 – Anpassung der ICD an das 21. Jahrhundert. Bundesgesundheitsblatt.

[23] Tanno LK, Chalmers RJ, Calderon MA, Aymé S, Demoly P, the Joint Allergy Academies (2017). Reaching multidisciplinary consensus on classification of anaphylaxis for the eleventh revision of the World Health Organization’s (WHO) International Classification of Diseases (ICD-11). Orphanet J Rare Dis.

[24] Tanno LK, Molinari N, Bruel S, Bourrain IL, Calderon M, Aubas P, Demoly P, the Joint Allergy Academies (2017). Field-testing the new anaphylaxis’ classification for the WHO International Classification of Diseases-11 revision. Allergy.

[25] Worm M, Moneret-Vautrin A, Scherer K, Lang R, Fernandez-Riva M, Cardona V (2014). First European data from the network of severe allergic reactions (NORA). Allergy.

[26] Worm M, Sturm G, Kleine-Tebbe J, Cichocka-Jarosz E, Cardona V, Maris I, Dölle S (2017). New trends in anaphylaxis. Allergo J Int.

[27] Worm M, Edenharter G, Ruëff F, Scherer K, Pföhler C, Mahler V (2012). Symptom profile and risk factors of anaphylaxis in Central Europe. Allergy.

[28] Worm M, Francuzik W, Renaudin JM, Bilò MB, Cardona V, Scherer Hofmeier K (2018). Factors increasing the risk for a severe reaction in anaphylaxis: An analysis of data from The European Anaphylaxis Registry. Allergy.

[29] Ring J, Messmer K (1977). Incidence and severity of anaphylactoid reactions to colloid volume substitutes. Lancet.

[30] Smith PL, Kagey-Sobotka A, Bleecker ER, Traystman R, Kaplan AP, Gralnick H (1980). Physiologic manifestations of human anaphylaxis. J Clin Invest.

[31] Reber LL, Hernandez JD, Galli SJ (2017). The pathophysiology of anaphylaxis. J Allergy Clin Immunol.

[32] Kalesnikoff J, Galli SJ (2010). Anaphylaxis: mechanisms of mast cell activation. Chem Immunol Allergy.

[33] Vadas P, Perelman B, Liss G (2013). Platelet-activating factor, histamine, and tryptase levels in human anaphylaxis. J Allergy Clin Immunol.

[34] Lee JK, Vadas P (2011). Anaphylaxis: mechanisms and management. Clin Exp Allergy.

[35] Kaliner M, Sigler R, Summers R, Shelhamer JH (1981). Effects of infused histamine: analysis of the effects of H-1 and H-2 histamine receptor antagonists on cardiovascular and pulmonary responses. J Allergy Clin Immunol.

[36] Vigorito C, Russo P, Picotti GB, Chiariello M, Poto S, Marone G (1983). Cardiovascular effects of histamine infusion in man. J Cardiovasc Pharmacol.

[37] Ring J (2004). Angewandte Allergologie.

[38] Finkelman FD, Khodoun MV, Strait R (2016). Human IgE-independent systemic anaphylaxis. J Allergy Clin Immunol.

[39] Solinski HJ, Gudermann T, Breit A (2014). Pharmacology and signaling of MAS-related G Protein coupled receptors. Pharmacol Rev.

[40] Ruëff F, Przybilla B, Bilò MB, Müller U, Scheipl F, Aberer W (2009). Predictors of severe systemic anaphylactic reactions in patients with Hymenoptera venom allergy: importance of baseline serum tryptase—a study of the European Academy of Allergology and Clinical Immunology Interest Group on Insect Venom Hypersensitivity. J Allergy Clin Immunol.

[41] Brockow K, Jofer C, Behrendt H, Ring J (2008). Anaphylaxis in patients with mastocytosis: a study on history, clinical features and risk factors in 120 patients. Allergy.

[42] Guenova E, Volz T, Eichner M, Hoetzenecker W, Caroli U, Griesinger G (2010). Basal serum tryptase as risk assessment for severe Hymenoptera sting reactions in elderly. Allergy.

[43] Schuch A, Brockow K (2017). Mastocytosis and anaphylaxis. Immunol Allergy Clin North Am.

[44] Przybilla B, Ruëff F, Walker A, Räwer HC, Aberer W, Bauer CP et al. Diagnose und Therapie der Bienen- und Wespengiftallergie. Leitlinie der Deutschen Gesellschaft für Allergologie und klinische Immunologie (DGAKI), des Ärzteverbandes Deutscher Allergologen (ÄDA), der Gesellschaft für Pädiatrische Allergologie und Umweltmedizin (GPA), der Deutschen Dermatologischen Gesellschaft (DDG) und der Deutschen Gesellschaft für Kinder- und Jugendmedizin (DGKJ) in Zusammenarbeit mit der Österreichischen Gesellschaft für Allergologie und Immunologie (ÖGAI) und der Schweizerischen Gesellschaft für Allergologie und Immunologie (SGAI). Allergo J 2011;20:318–39

[45] De Schryver S, Halbrich M, Clarke A, La Vieille S, Eisman H, Alizadehfar R (2016). Tryptase levels in children presenting with anaphylaxis: temporal trends and associated factors. J Allergy Clin Immunol.

[46] Sturm GJ, Varga EM, Roberts G, Mosbech H, Bilò MB, Akdis CA (2018). EAACI Guidelines on allergen immunotherapy: hymenoptera venom allergy. Allergy.

[47] Stark BJ, Sullivan TJ (1986). Biphasic and protracted anaphylaxis. J Allergy Clin Immunol.

[48] Kim TH, Yoon SH, Lee SY, Choi YH, Park CM, Kang HR (2018). Biphasic and protracted anaphylaxis to iodinated contrast media. Eur Radiol.

[49] Rohacek M, Edenhofer H, Bircher A, Bingisser R (2014). Biphasic anaphylactic reaction: occurrene and mortality. Allergy.

[50] Lieberman P (2005). Biphasic anaphylactic reactions. Ann Allergy Astma Immunol.

[51] Commins SP, Platts-Mills TA (2009). Anaphylaxis syndromes related to a new mammalian cross-reactive carbohydrate determinant. J Allergy Clin Immunol.

[52] Fischer J, Lupberger E, Hebsaker J, Blumenstock G, Aichinger E, Yazdi AS (2017). Prevalence of type I sensitization to alpha-gal in forest service employees and hunters. Allergy.

[53] Weins AB, Eberlein B, Biedermann T (2019). Diagnostics of alpha-gal syndrome: current standards, pitfalls and perspectives. Hautarzt.

[54] Blümchen K, Beder A, Beschoner J, Ahrens F, Gruebl A, Hamelmann E (2014). Modified oral food challenge used with sensitization biomarkers provides more real-life clinical thresholds for peanut allergy. J Allergy Clin Immunol.

[55] Barnard JH (1973). Studies of 400 Hymenoptera sting deaths in the United States. J Allergy Clin Immunol.

[56] Delage C, Irey NS (1972). Anaphylactic deaths: a clinicopathologic study of 43 cases. J Forensic Sci.

[57] Fisher MM (1986). Clinical observations on the pathophysiology and treatment of anaphylactic cardiovascular collapse. Anaesth Intensive Care.

[58] Timmermann A, Böttiger BW, Byhahn C, Dörges V, Eich C. S1-Leitlinie (AMWF). Prähospitales Atemwegsmanagement. 02/2019.. https://www.awmf.org/uploads/tx_szleitlinien/001-040l_S1_Praehospitales-Atemwegsmanagement_2019-03_1.pdf. 10.19224/ai2019.316

[59] Pumphrey RS, Roberts IS (2000). Postmortem findings after fatal anaphylactic reactions. J Clin Pathol.

[60] Pumphrey R (2004). Anaphylaxis: can we tell who is at risk of a fatal reaction?. Curr Opin Allergy Clin Immunol.

[61] von Krogh G, Maibach HI (1981). The contact urticaria syndrome—an updated review. J Am Acad Dermatol.

[62] Willi R, Pfab F, Huss-Marp J, Buters JT, Zilker T, Behrendt H (2009). Contact anaphylaxis and protein contact dermatitis in a cook handling chicory leaves. Contact Derm.

[63] Fleischer DM, Greenhawt M, Sussman G, Bégin P, Nowak-Wegrzyn A, Petroni D (2019). Effect of epicutaneous immunotherapy vs placebo on reaction to peanut protein ingestion among children with peanut allergy: the PEPITES Randomized Clinical Trial. JAMA.

[64] Kemp SF, Lockey RF (2002). Anaphylaxis: a review of causes and mechanisms. J Allergy Clin Immunol.

[65] Morita E, Kunie K, Matsuo H (2007). Food-dependent exercise-induced anaphylaxis. J Dermatol Sci.

[66] Scherf KA, Brockow K, Biedermann T, Koehler P, Wieser H (2016). Wheat-dependent exercise-induced anaphylaxis. Clin Exp Allergy.

[67] Schwartz LB (2001). Clinical utility of tryptase levels in systemic mastocytosis and associated hematologic disorders. Leuk Res.

[68] Jakobs RL, Rake GW, Fournier DC, Chilton RJ, Culver WG, Beckmann CH (1981). Potentiated anaphylaxis in patients with drug-induced beta-adrenergic blockade. J Allergy Clin Immunol.

[69] Newman BR, Schultz LK (1981). Epinephrine-resistant anaphylaxis in a patient taking propranolol hydrochloride. Ann Allergy.

[70] Toogood JH (1988). Risk of anaphylaxis in patients receiving beta-blocker drugs. J Allergy Clin Immunol.

[71] Lang DM, Alpern MB, Visintainer PF, Smith ST (1991). Increased risk for anaphylactoid reaction from contrast media in patients on beta-adrenergic blockers or with asthma. Ann Intern Med.

[72] Lang DM, Alpern MB, Visintainer PF, Smith ST (1993). Elevated risk of anaphylactoid reaction from radiographic contrast media is associated with both beta-blocker exposure and cardiovascular disorders. Arch Intern Med.

[73] Mullins RJ, Dear KB, Tang ML (2009). Characteristics of childhood peanut allergy in the Australian Capital Territory, 1995 to 2007. J Allergy Clin Immunol.

[74] Pouessel G, Dumond P, Liabeuf V, Tanno LK, Deschildre A, Beaumont P (2019). Gaps in the management of food-induced anaphylaxis reactions at school. Pediatr Allergy Immunol.

[75] Ring J, Klimek L, Worm M (2018). Adrenalin in der Akutbehandlung der Anaphylaxie. Dtsch Arztebl Int.

[76] Westfall TC, Westfall DP, Hardman JG, Limbird LE, Goodman A (2002). Adrenergic agonists and antagonists: catecholamines and sympathomimetic drugs. Goodman & Gilmans The pharmacological basis of therapeutics.

[77] Nolan JP, Soar J, Zideman DA, Biarent D, Bossaert LL, Deakin C (2010). European Resuscitation Council Guidelines for Resuscitation 2010 Section 1. Executive summary. Resuscitation.

[78] Thomas M, Crawford I (2005). Best evidence topic report. Glucagon infusion in refractory anaphylactic shock in patients on beta-blockers. Emerg Med J.

[79] Simons FE, Schatz M (2012). Anaphylaxis during pregnancy. J Allergy Clin Immunol.

[80] Perkins GD, Olasveengen TM, Maconochie I, Soar J, Wyliie J, Greif R et al. European Resuscitation Council guidelines for resuscitation: 2017 update. Resuscitation. 2018;123:43–50. 10.1016/j.resuscitation.2017.12.007 29233740

[81] Monsieurs KG, Nolan JP, Bossaert LL, Greif R, Maconochie IK, Nikolaou NI (2015). European Resuscitation Council guidelines for resuscitation 2015: Section 1. executive summary. Resuscitation.

[82] Bellomo R, Chapman M, Finfer S, Hickling K, Myburgh J (2000). Low-dose dopamine in patients with early renal dysfunction: a placebo-controlled randomised trial. Australian and New Zealand Intensive Care Society (ANZICS) Clinical Trials Group. Lancet.

[83] Friedrich JO, Adhikari N, Herridge MS, Beyene J (2005). Meta-analysis: low-dose dopamine increases urine output but does not prevent renal dysfunction or death. Ann Intern Med.

[84] Gronemeyer W (1980). Noradrenalin statt Adrenalin beim anaphylaktischen Schock. Dtsch Med Wochenschr.

[85] Schummer C, Wirsing M, Schummer W (2008). The pivotal role of vasopressin in refractory anaphylactic shock. Anesth Analg.

[86] Messmer K, Tinker J, Rapin M (1983). Plasma substitutes and indications for their use. Care of the critically ill patient.

[87] Stoelting RK, Stoelting RK (2006). Systemic circulation. Pharmacology & physiology in anesthetic practice.

[88] Walter A, Böttiger BW (2004). Anaphylaktoide Reaktionen in der Prähospitalphase. Internist.

[89] Vincent JL, De Backer D (2013). Circulatory shock. N Engl J Med.

[90] Martin C, Jacob M, Vicaut E, Guidet B, Van Aken H, Kurz A (2013). Effect of waxy maize-derived hydroxyethyl starch 130/0.4 on renal function in surgical patients. Anesthesiology.

[91] Myburgh JA, Finfer S, Bellomo R, Billot L, Cass A, Gattas D, CHEST Investigators, Australian and New Zealand Intensive Care Society Clinical Trials Group (2012). Hydroxyethyl starch or saline for fluid resuscitation in intensive care. N Engl J Med.

[92] Bundesärztekammer rote Hand Brief. HES solutions for infusion 13-08-2018

[93] Sheikh A, Ten Broek V, Brown SG, Simons FE (2007). H1-antihistamines for the treatment of anaphylaxis: Cochrane systematic review. Allergy.

[94] Pragst F, Herre S, Bakdash A (2006). Poisonings with diphenhydramine—a survey of 68 clinical and 55 death cases. Forensic Sci Int.

[95] Zuberbier T, Asero R, Bindslev-Jensen C, Canonica WG, Church MK, Giménez-Arnau AM, Dermatology Section of the European Academy of Allergology and Clinical Immunology, Global Allergy and Asthma European Network, European Dermatology Forum; World Allergy Organization (2009). EAACI/GA(2)LEN/EDF/WAO guideline: management of urticaria. Allergy.

[96] Lin RY, Curry A, Pesola GR, Knight RJ, Lee HS, Bakalchuk L (2000). Improved outcomes in patients with acute allergic syndromes who are treated with combined H1 and H2 antagonists. Ann Emerg Med.

[97] Ring J, Rothenberger KH, Clauss W (1985). Prevention of anaphylactoid reactions after radiographic contrast media infusion by combined histamine H1- and H2-receptor antagonists: results of a prospective controlled trial. Int Arch Allergy Appl Immunol.

[98] Brockow K, Kiehn M, Riethmüller C, Vieluf D, Berger J, Ring J (1997). Efficacy of antihistamine pretreatment in the prevention of adverse reactions to Hymenoptera immunotherapy: a prospective, randomized, placebo-controlled trial. J Allergy Clin Immunol.

[99] Aouam K, Bouida W, Fredj BN, Chaabane A, Boubaker H, Boukef R (2012). Severe ranitidine-induced anaphylaxis: a case report and literature review. J Clin Pharm Ther.

[100] Winbery SL, Lieberman PL (2002). Histamine and antihistamines in anaphylaxis. Clin Allergy Immunol.

[101] Choo KJ, Simons E, Sheikh A (2010). Glucocorticoids for the treatment of anaphylaxis: Cochrane systematic review. Allergy.

[102] Alqurashi W, Ellis AK (2017). Do corticosteroids prevent biphasic anaphylaxis?. J Allergy Clin Immunol Practic.

[103] Fischer J, Biedermann T (2009). Anaphylaxie auf allergologische Testung oder Therapie – ein Handlungsleitfaden zum Notfallmanagement. Allergo J.

[104] Bernhard M, Hossfeld B, Bein B, Böttiger BW, Bohn A, Fischer M, Gräsner JT, et al. https://www.awmf.org/uploads/tx_szleitlinien/001-030l_S1_Praehospitale_Notfallnarkose_Erwachsene_2015-03-verlaengert.pdf. Handlungsempfehlung zur Praehospitalen Notfallnarkose bei Erwachsenen Anaesthesie Intensivmed. 2015;56:317–335.

[105] Lommatzsch M, Buhl R, Korn S. The treatment of mild and moderate asthma in adults. Dtsch Aerztebl Int. 2020;117:434–444 10.3238/arztebl.2020.0434 PMC7490458 32885783

[106] Lieberman JA, Chehade M (2013). Use of omalizumab in the treatment of food allergy and anaphylaxis. Curr Allergy Asthma Rep.

[107] Simons FE, Ardusso LR, Dimov V, Ebisawa M, El-Gamal YM, Lockey RF, World Allergy Organization (2013). World Allergy Organization Anaphylaxis Guidelines: 2013 update of the evidence base. Int Arch Allergy Immunol.

[108] Beyer K, Niggemann B (2016). Food allergy in childhood. Bundesgesundheitsblatt.

[109] Beyer K, Grabenhenrich L, Härtl M, Beder A, Kalb B, Ziegert M (2015). Predictive values of component-specific IgE for the outcome of peanut and hazelnut food challenges in children. Allergy.

[110] Worm M, Reese I, Ballmer-Weber B, Beyer K, Bischoff SC, Classen M (2015). Guidelines on the management of IgE-mediated food allergies. S2k-Guidelines of the German Society for Allergology and Clinical Immunology (DGAKI) in collaboration with the German Medical Association of Allergologists (AeDA), the German Professional Association of Pediatricians (BVKJ), the German Allergy and Asthma Association (DAAB), German Dermatological Society (DDG), the German Society for Nutrition (DGE), the German Society for Gastroenterology, Digestive and Metabolic Diseases (DGVS), the German Society for Oto-Rhino-Laryngology, Head and Neck Surgery, the German Society for Pediatric and Adolescent Medicine (DGKJ), the German Society for Pediatric Allergology and Environmental Medicine (GPA), the German Society for Pneumology (DGP), the German Society for Pediatric Gastroenterology and Nutrition (GPGE), German Contact Allergy Group (DKG), the Austrian Society for Allergology and Immunology (Æ-GAI), German Professional Association of Nutritional Sciences (VDOE) and the Association of the Scientific Medical Societies Germany (AWMF). Allergo J Int.

[111] Lange L, Lasota L, Finger A, Vlajnic D, Büsing S, Meister J (2017). Ana o 3-specific IgE is a good predictor for clinically relevant cashew allergy in children. Allergy.

[112] Guerlain S, Hugine A, Wang L (2010). A comparison of 4 epinephrine autoinjector delivery systems: usability and patient preference. Ann Allergy Asthma Immunol.

[113] Ring J, Beyer K, Dorsch A, Biedermann T, Fischer J, Friedrichs F (2012). Anaphylaxieschulung – ein neues Behandlungsprogramm zur tertiären Krankheitsprävention nach Anaphylaxie. Allergo J.

[114] Bilò MB, Cichocka-Jarosz E, Pumphrey R, Oude-Elberink JN, Lange J, Jakob T (2016). Self-medication of anaphylactic reactions due to hymenoptera stings—an EAACI Task Force Consensus Statement. Allergy.

[115] Brockow K, Schallmayer S, Beyer K, Biedermann T, Fischer J, Gebert N (2015). Effects of a structured educational intervention on knowledge and emergency management in patients at risk for anaphylaxis. Allergy.

[116] Ring J, Brockow K, Kugler C, Gebert N, Grando K, Götz D (2017). Neue Aspekte zur Allergie-Edukation: Beispiel Anaphylaxie. Allergo J.

[117] Worm M, Ring J, Klimek L, Jakob T, Lange L, Treudler R, et al. Anaphylaxie-Risiko bei der COVID-19-Impfung – Empfehlungen für das praktische Management. MMW Fortschr Med 2021;163:48–51. (in press). 10.1007/s15006-021-9530-6 PMC7814269 33464512

[118] Klimek L, Novak N, Worm M, Hamelmann E, Werfel T, Wagenmann M, et al. AeDA/DGAKI/GPA-Stellungnahme zu schweren allergischen Reaktionen nach COVID-19-Impfung mit dem Impfstoff von Pfizer/BioNTech in Großbritannien. Allergo J Int. 2021. 10.1007/s40629-020-00160-4. 10.1007/s15007-021-4762-4 PMC7994061 33785990

